# SRSF10 stabilizes CDC25A by triggering exon 6 skipping to promote hepatocarcinogenesis

**DOI:** 10.1186/s13046-022-02558-0

**Published:** 2022-12-20

**Authors:** Xiaoming Liu, Yongqiang Zheng, Mengqing Xiao, Xingyu Chen, Yuxing Zhu, Canxia Xu, Fen Wang, Zexian Liu, Ke Cao

**Affiliations:** 1 Department of Oncology, Third Xiangya Hospital of Central South University, Changsha, 410013 China; 2 Department of Gastroenterology, Third Xiangya Hospital of Central South University, Changsha, 410013 China; 3 State Key Laboratory of Oncology in South China, Collaborative Innovation Center for Cancer Medicine, Sun Yat-Sen University Cancer Center, 510060 Guangzhou, China

**Keywords:** SRSF10, CDC25A, Alternative splicing, Exon skipping, Hepatocellular carcinoma

## Abstract

**Background:**

Alternative splicing (AS) events are extensively involved in the progression of diverse tumors, but how serine/arginine-rich splicing Factor 10 (SRSF10) behaves in hepatocellular carcinoma (HCC) has not been sufficiently studied. We aimed to determine SRSF10 associated AS mechanisms and their effects on HCC progression.

**Methods:**

The expression of SRSF10 in HCC tissues was examined, and the in vitro and in vivo functions of SRSF10 were investigated. The downstream AS targets were screened using RNA sequencing. The interaction between SRSF10 protein and exclusion of cell division cycle 25 A (CDC25A) mRNA was identified using RNA immunoprecipitation and crosslinking immunoprecipitation q-PCR. The effects of SRSF10 on CDC25A posttranslational modification, subcellular distribution, and protein stability were verified through coimmunoprecipitation, immunofluorescence, and western blotting.

**Results:**

SRSF10 was enriched in HCC tissues and facilitated HCC proliferation, cell cycle, and invasion. RNA sequencing showed that SRSF10 promotes exon 6 exclusion of CDC25A pre-mRNA splicing. As a crucial cell cycle mediator, the exon-skipped isoform CDC25A(△E6) was identified to be stabilized and retained in the nucleus due to the deletion of two ubiquitination (Lys150, Lys169) sites in exon 6. The stabilized isoform CDC25A(△E6) derived from AS had stronger cell cycle effects on HCC tumorigenesis, and playing a more significant role than the commonly expressed longer variant CDC25A(L). Interestingly, SRSF10 activated the carcinogenesis role of CDC25A through Ser178 dephosphorylation to cause nuclear retention. Moreover, CDC25A(△E6) was verified to be indispensable for SRSF10 to promote HCC development in vitro and in vivo.

**Conclusions:**

We reveal a regulatory pattern whereby SRSF10 contributes to a large proportion of stabilized CDC25A(△E6) production, which is indispensable for SRSF10 to promote HCC development. Our findings uncover AS mechanisms such as CDC25A that might serve as potential therapeutic targets to treat HCC.

**Supplementary Information:**

The online version contains supplementary material available at 10.1186/s13046-022-02558-0.

## Introduction

Liver cancer tumors are among the five most lethal solid tumors in China and the third most lethal neoplasms worldwide. Hepatocellular carcinoma (HCC) accounts for more than 80% of all liver cancer cases, and the common difficulties in most cases are early diagnosis, the aggressive invasiveness of tumors, and the lack of a favorable response to pharmacotherapy [1]. Aberrant epigenetic regulation, posttranscriptional events, and posttranslational modifications (PTMs) are very common in HCC cells, among which the alternative splicing (AS) of pre-mRNA is a widespread posttranscriptional event in cells contributing to proteome diversity [2]. Current investigations are focused on better understanding disease-relevant molecular mechanisms, such as AS [3], which is indispensable for novel targeted therapy.

Deregulation of AS is implicated as a relevant source of molecular heterogeneity in cancer. Editing of immature mRNA (pre-mRNA), “constitutive splicing” is carried out by the spliceosome. This process is a fundamental step for intron removal and exon ligation as part of an integrated mechanism involved in the gene regulatory network [4]. In contrast, the “cut-and-paste” process in AS generates diverse forms of partial or complete removal or retention of exons and introns precisely because of different splicing sites, which is understood to bring about structurally and functionally dissimilar splicing end-products [5]. These products play important roles in the regulation of cancer cell activities, including sustaining proliferative signaling, evading growth suppression, resisting cell death, triggering invasion and metastasis, and deregulating cellular energetics [6, 7]. Aberrant splicing generates mRNA isoforms involved in liver cancer development and malignancy [8]. The relationship between AS signatures and the prognosis of HCC has been investigated by several studies [9, 10], but the impact of these variants on liver cancer biology remains largely unknown.

The sequential interaction of spliceosome elements such as small nuclear ribonucleoproteins (snRNPs) is affected by the balance between members of serine/arginine (SR)-rich proteins and heterogeneous nuclear ribonucleoproteins (hnRNPs), which determines the outcome of mRNA maturation [11, 12]. Among the splicing factors involved in this process are SR proteins, which are characterized by a domain rich in arginine/serine dipeptides, favoring short alternatives of splicing [13, 14]. In contrast, hnRNPs bind to exonic or intronic splicing silencers (ESS or ISS), inhibiting short splicing options and hence favoring the formation of long isoforms [15]. Considering the important roles of SR proteins together with other spliceosome components, it is interesting to note their global profile of alternative RNA splicing events to determine various biological effects. Specifically, a general downregulation of SRSF1, SRSF3, SRSF5, and SRSF9 has been found in HCC [16]. However, whether SR family members can act as oncogenes or tumor suppressors remains unclear. For instance, SRSF2 protein, could act as either an HCC driver or suppressor due to its species-specific characteristic [17, 18]. These observations led us to investigate the hypothesis that dysregulated SR proteins may result in HCC tumorigenesis and progression.

To date, the potential functions of SRSF10 in tumorigenesis are undocumented for most cancer types, and its pathological mechanisms are poorly characterized. In our previous study, SRSF10 was found to be significantly upregulated in HCC tumorigenesis, but how SRSF10 behaves in HCC is not clear. Our initial observations indicate that SRSF10 overexpression stimulates HCC cell growth and invasion. Intriguingly, SRSF10 has an essential role in affecting several AS events, such as downstream cell division cycle 25 A (CDC25A) proteins. The following data collectively indicate that CDC25A regulated by SRSF10 contributes to HCC progression, thus identifying the AS of CDC25A, such as exon skipping, as a potential therapeutic target in HCC.

## Materials and methods

### Cell lines and cell culture

Six HCC cell lines (Huh7, HepG2, Hep3B, MHCC97H, MHCC97L, and MHCC-LM3) were purchased from ATCC (Rockville, Maryland). Cells were continuously cultured in Dulbecco’s modified Eagle’s medium (Sigma–Aldrich, USA) containing 10% fetal bovine serum (FBS) (Gibco, New York, USA), 100 IU/ml penicillin G and 100 μg/ml streptomycin (Sigma–Aldrich, USA). All cell lines were incubated at 37 °C in a humidified atmosphere with 5% CO_2_.

### Plasmid construction, RNA interference and transfection

SRSF10-shRNA, plasmids for SRSF10 overexpression with and without Flag-tag, CDC25A-overexpressing plasmids tagged with and without GFP-Flag, and CDC25A-shRNA targeting various sites were purchased from GeneChem Biotechnology Company (Shanghai, China). The shRNA sequences and primer information are described in more detail in Supplementary Table 1.

The 24/48 h transfection and knockdown efficiency were assessed by immunofluorescence and western blot analysis. The knockdown efficiency was determined using two different RNAi, and the sequence with the higher result was selected for further experiments.

### qRT–PCR and RNA sequencing

Total RNA was extracted from cells using an RNeasy Mini kit (Invitrogen, Life Technologies, Carlsbad, CA, USA). Complementary DNA (cDNA) synthesis was performed with a GeneAmp RNA PCR kit (Life Technologies) using 1 µg RNA per sample. qPCRs were set up using the iScriptTM two-step RT–PCR kit with SYBR Green (Invitrogen) and the targeting gene primers (see Supplementary Table 1).

RNA array was performed on an Illumina HiSeq machine with paired-end sequencing under the support of Kangchen Biotech (200,233; Shanghai, China).

### Reagents and antibodies

The chemical reagents rabusertib (CHK1-specific antagonist also known as LY2603618; cat. #s2626), and MG132 (proteasome inhibitor; cat. #s2619) were obtained from Selleckchem (Houston, TX, USA). CHX (cycloheximide, protein synthesis inhibitor; cat. #2112 s) was obtained from Cell Signaling Technology (Beverly, Massachusetts, USA). Commercially available antibodies for western blotting, immunoprecipitation, immunohistochemistry, and immunofluorescence staining are described in more detail in Supplementary Table 2.

### Mutagenesis

Mutation and truncation of SRSF10 and CDC25A were performed by PCR-based methods. The CDSs of SRSF10 (NM_054016.4) and CDC25A (NM_001789) were synthesized and subcloned into the vector pEGFP-C1 (Clontech, 5' BglII—3' BamHI). Point mutation of CDC25A in Lys150 and Lys169 was introduced to arginine (R), while its Ser178 was replaced by alanine (A) or glutamic acid (E). For SRSF10, deleting the 11–84 aa sequence led to a mutation lacking the RNA recognition motif (RRM), also known as SRSF10 (ΔRRM1). Both SRSF10 and CDC25A wild-type and mutants were validated by DNA sequencing and western blotting with the help of Kangchen Biotech (200,233; Shanghai, China).

### Cell proliferation, cell cycle, and invasion assays

Cell proliferation was analyzed using a commercial CCK-8 assay kit (#C0038, Beyotime) and EdU detection kit (#C0075S, Beyotime). Fluorescence-activated cell sorting (FACS) was used to assess the cell cycle with a PI staining kit (#R40432, Sigma). Cell invasion was assessed by the transwell assay with a 6-well insert device (8 μm pore size; Corning Life Sciences, Bedford, MA) and Biocoat Matrigel (BD Biosciences) according to the manufacturer’s instructions.

### Cycloheximide (CHX)-based protein stability assay

Cells were treated with 10 μM cycloheximide (CHX) for the indicated periods (0 h, 1 h, 2 h, 4 h, and 8 h) to block protein synthesis. MG132 (20 μM) was also administered to inhibit the proteasome before harvesting. Endogenous and exogenous CDC25A protein expression was then assayed as described previously [19].

### mRNA decay assay

To measure mRNA stability, 5 g/ml actinomycin D (Sigma Aldrich, USA) was added to cells to inhibit transcription, followed by incubation for the different times indicated. Total RNA was extracted at each time point and quantitated by RT‒PCR. The transcript levels were plotted to create the appropriate nonlinear regression curves using a one-phase decay equation. Exponential fitting curves were determined to quantify the RNA decay rate constant (y = a*e^−kt^; where k is the decay rate constant, y is the relative amount of RNA, and t is the time). The rate of mRNA turnover was estimated according to the half-life t_1/2_ = ln(2)/k. The transcript 18S rRNA, which does not decay over time, was detected as a control.

### Protein extraction, immunoprecipitation and western blot analysis

Protein extracts were obtained by washing the cells once with PBS and resuspending them in lysis buffer. Tissues were lysed using a cell disruptor and a complete protease inhibitor cocktail (Cat. #5,056,489,001, Roche).

Immunoprecipitation was performed using protein G-agarose (Millipore, Temecula, CA). Blots were then developed with ECL western blotting reagents (Pierce Biotechnology, Rockford, IL). The signal intensity was quantified with ImageJ (National Institutes of Health, Bethesda, MD).

### Immunohistochemistry

Tissues were obtained from the specimens of HCC patients who met the following criteria: 1) informed consent was provided; 2) the patients were clinically and pathologically diagnosed with HCC; 3) the patients did not undergo any neoadjuvant therapy before surgery; and 4) adjacent nonneoplastic liver tissues were available for comparison. A total of 74 paired HCC specimens embedded in paraffin were cut into 5-µm slides. The sections were counterstained as previously described [20].

The degree of immunostaining was reviewed and scored separately by two independent pathologists who were blinded to the histopathological features of the samples. Based on the proportion of tumor cells, the following scores were assigned: 0, no tumor cells; 1, < 10% tumor cells; 2, 10 − 35% tumor cells; 3, 35 − 75% tumor cells; and 4, > 75% tumor cells. The staining intensity was scored as follows: 1, no staining; 2, weakly stained (light yellow); 3, moderately stained (yellow brown); and 4, strongly stained (brown). The staining index was determined by multiplying the staining intensity score by the tumor cell proportion score. A staining index ≥ 6 was considered to indicate high expression of SRSF10, while an index < 6 indicated low expression.

### Immunofluorescence staining

Cells grown on coverslips were stained as previously described [20]. The antibodies used are listed in Supplementary Table 2. Images were obtained using a confocal microscope (TCSSP8, Leica Microsystems) equipped with an acousto-optical beam splitter, a 405-nm laser (for DAPI), an argon laser (488 nm for Alexa 488), and a diode-pumped solid-state (DPSS) laser (561 nm).

### RNA immunoprecipitation (RIP) and crosslinking immunoprecipitation q-PCR (CLIP-qPCR)

Immunoprecipitation targeting CDC25A pre-mRNA was performed using Magna RIP™ (Catalog No. 17–700, MilliporeSigma, USA) according to the manufacturer’s protocol. The SRSF10 antibody or IgG-immunoprecipitated RNA extracts were reverse-transcribed using TRIzol Reagent (Invitrogen, Carlsbad, CA, USA), and the quantification of target RNA was performed by RT‒qPCR).

In the CLIP study of Flag-SRSF10, we tested the recognition of CDC25A. Three primer pairs for exon 5, exon 6, and exon 7 were designed to evaluate precipitated RNA levels as previously described [21]. The sequences of these primers are presented in more detail in Supplementary Table 1.

### Tumor xenograft models

Five-week-old male BALB/c nude mice were randomly divided into six groups. Specifically, xenograft tumors were generated by subcutaneously inoculating the right axillary fossa with 200 μl (1 × 10^6^ cells) of SRSF10, CDC25A(△E6), CDC25A(L), sh-CDC25A(△E6), or sh-CDC25A(FL) in HepG2 cells and control cells. The size of the palpable tumors was recorded every 3 days by measuring the tumor length (L, the longest diameter) and width (W, the shortest diameter), which were recorded six consecutive times. All mice were sacrificed after 35 days. The tumor volume (V) was calculated according to the formula V = 1/2 (L × W^2^).

### Bioinformatic analyses

The survival, differential expression, and correlation of the candidate gene were assessed using the Gene Expression Profiling Interactive Analysis (GEPIA) database (http://gepia.cancer-pku.cn), the starBase Pan-Cancer Analysis Platform (http://starbase.sysu.edu.cn/panCancer.php), the Cancer Genome Atlas (TCGA) database (https://cancergenome.nih.gov/), Cancer RNA-Seq Nexus (CRN) database (http://syslab4.nchu.edu.tw/), and NCBI’s Gene Expression Omnibus database (https://www.ncbi.nlm.nih.gov/geo/). PhosphoSitePlus (https://www.phosphosite.org/) was applied to predict the PTM sites. The Ensembl Genome Browser (https://www.ensembl.org/) was used to blast RNA sequences of CDC25A variants. Gene set enrichment analysis (GSEA) of SRSF10- and CDC25A-relevant gene signatures was performed with GSEA software v.2.0 according to a reported protocol [22].

### Statistical analyses

Statistical analyses were performed using SPSS 19.0 software (version 19.0; SPSS Inc., Chicago, IL). The results are displayed as the mean ± SD or mean ± SEM, and two-group comparisons were evaluated using Student’s t test.

The relationship between SRSF10 expression and clinicopathological characteristics was analyzed using the χ^2^ test. On the basis of the correlation between the IHC score and patient survival, the cutoff point of each dataset subgroup was determined using the survminer R package. The “surv-cut point” function, which repeatedly tested all potential cut points to find the maximum rank statistic, was applied to the IHC score. Patients were then divided into high- and low-score groups on the basis of maximally selected log-rank statistics to reduce the batch effect of calculation. In the univariate survival analysis, cumulative survival curves were calculated according to the Kaplan–Meier method, and the survival curves were analyzed using the log-rank test.

## Results

### SRSF10 is enriched in HCC and leads to malignant phenotypes

**Fig. 1:**
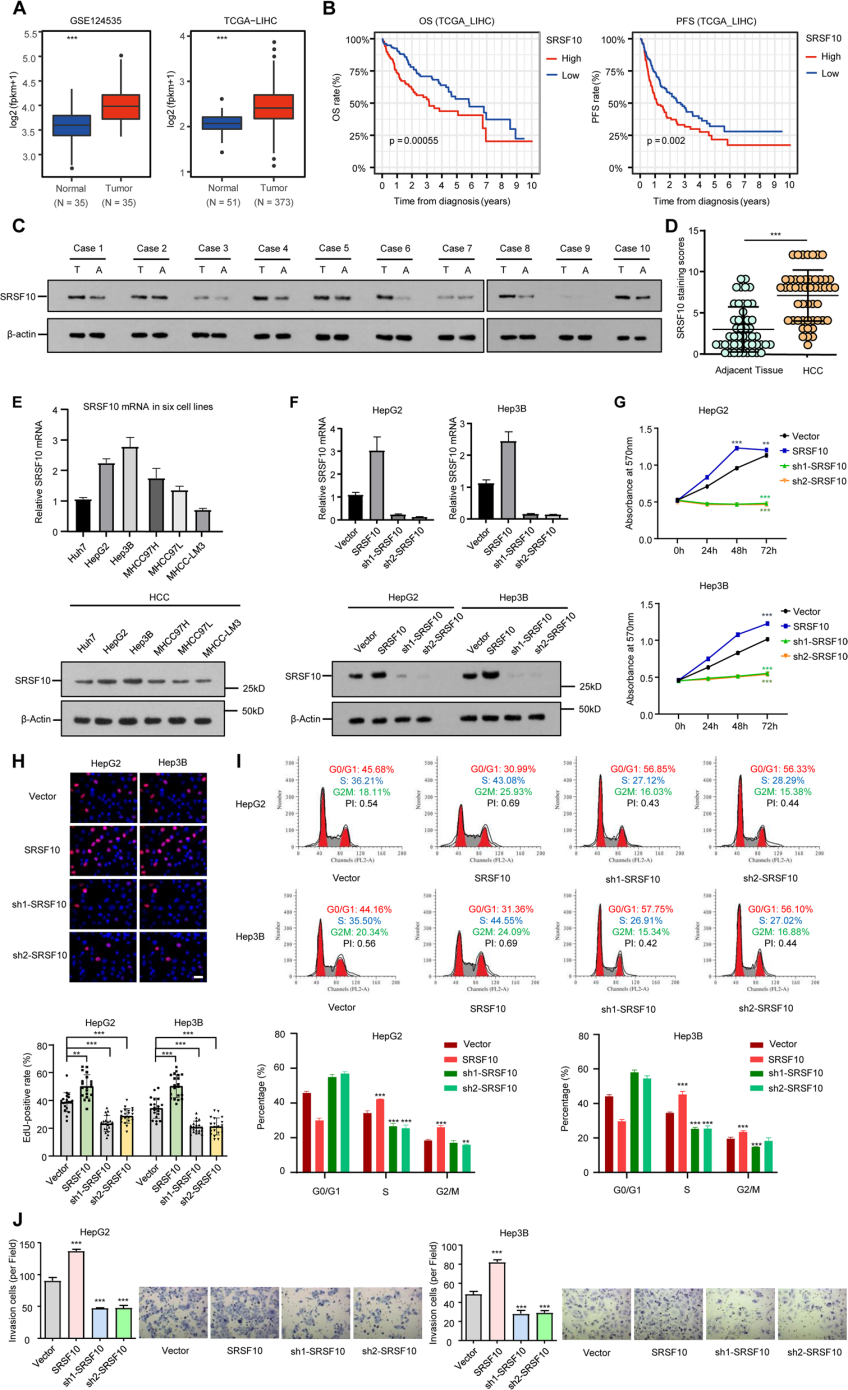
SRSF10 is enriched in HCC and stimulates tumor progression. **A** Comparison of SRSF10 mRNA levels between HCC and adjacent nontumor tissues from the GEO and TCGA databases. ***p < 0.001, t test. The expressional tendencies of SRSF10 from the GEO database are shown in Supplemental Fig. 1. **B** TCGA survival curve showing that SRSF10 expression was positively associated with shorter overall survival (OS) and progression-free survival (PFS) in HCC patients (n = 373). **C** Western blotting of SRSF10 levels in HCC tissue and matched nontumor tissue. Ten paired samples from our 74 selected patients are shown. **D** Immunohistochemical staining score of SRSF10 in HCC tissue and matching adjacent nonneoplastic tissue. ***p < 0.001, χ2 test. Ten paired images are displayed in Supplemental Fig. 2. **E** The mRNA (upper panel) and protein expression (lower panel) levels of SRSF10 in 6 HCC cell lines. β-actin was used as the loading control. **F** RT‒qPCR (upper panel) and western blotting (lower panel) of SRSF10 in response to different overexpression plasmids and shRNAs. sh2-SRSF10, which has a relatively high knockdown efficiency, was selected for further experiments. **G**, **H**, **I**, **J** Cell viability, proliferation, cycle, and invasion in response to SRSF10 alteration. **G** CCK-8 assays were conducted after 24 h, 48 h, and 72 h to test cell viability. **H** Data from EdU-positive cells showed DNA replication ability in the S phase. **I** Flow cytometry results of the percentages of cells in the G0/G1, S, and G2/M phases. **J** The invasion abilities were determined by transwell assay. Data are presented as the mean ± SD value of three biological replicates. **p < 0.01 and ***p < 0.001 vs. vector, t test. Scale bars, 20 μm

Analysis of the multiple Gene Expression Omnibus (GSE124535, normal (35) vs. tumor (35)) and TCGA datasets (normal (51) vs. tumor (373)) revealed significantly enriched SRSF10 in HCC versus normal tissue. SRSF10 was also associated with a shorter overall survival rate in HCC patients from public data (Fig. 1A, B, and Supplementary Fig. 1). These results are in line with previous proteomic assays in which SRSF10 was abundant in SIRT1-mediated HCC tumorigenesis [20]. In the current study, SRSF10 expression was detected in liver tissues of HCC patients, and the correlation of SRSF10 levels with tumor phenotypes was analyzed. The immunohistochemistry and western blot results (Fig. 1C, D, and Supplementary Fig. 2) indicate that HCC tissues had augmented SRSF10 compared with adjacent nontumor tissues. Multiple clinicopathological parameters reveal that SRSF10 in the HCC tissues (*n* = 74) was highly correlated with the neoplastic grade (Edmondson-Steiner, *P* = 0.01) and tumor size (*P* < 0.001) (see Supplementary Table 3). Moreover, bioinformatics revealed that 5 key genes involved in proliferation and metabolism were positively correlated with SRSF10 expression (see Supplementary Fig. 3). To investigate the expression of the SRSF10 gene in more detail, we measured the levels of SRSF10 products in HCC cells relative to control cells. As they have higher endogenous expression of SRSF10, HepG2 and Hep3B cells were selected as tool cell lines in the following interference experiments (Fig. 1E, F). According to the results of CCK-8, EdU, flow cytometry, and transwell assays, SRSF10 overexpression caused increased proliferation, cell cycle, and invasion in both Hep3B and HepG2 cells, while SRSF10 deletion had the opposite effects (Fig. 1G, H, I, and J). These data collectively indicate that a high level of SRSF10 positively correlates with HCC malignant phenotypes.

### SRSF10 promotes alternative splicing of pre-CDC25A through exon 6 exclusion

**Fig. 2:**
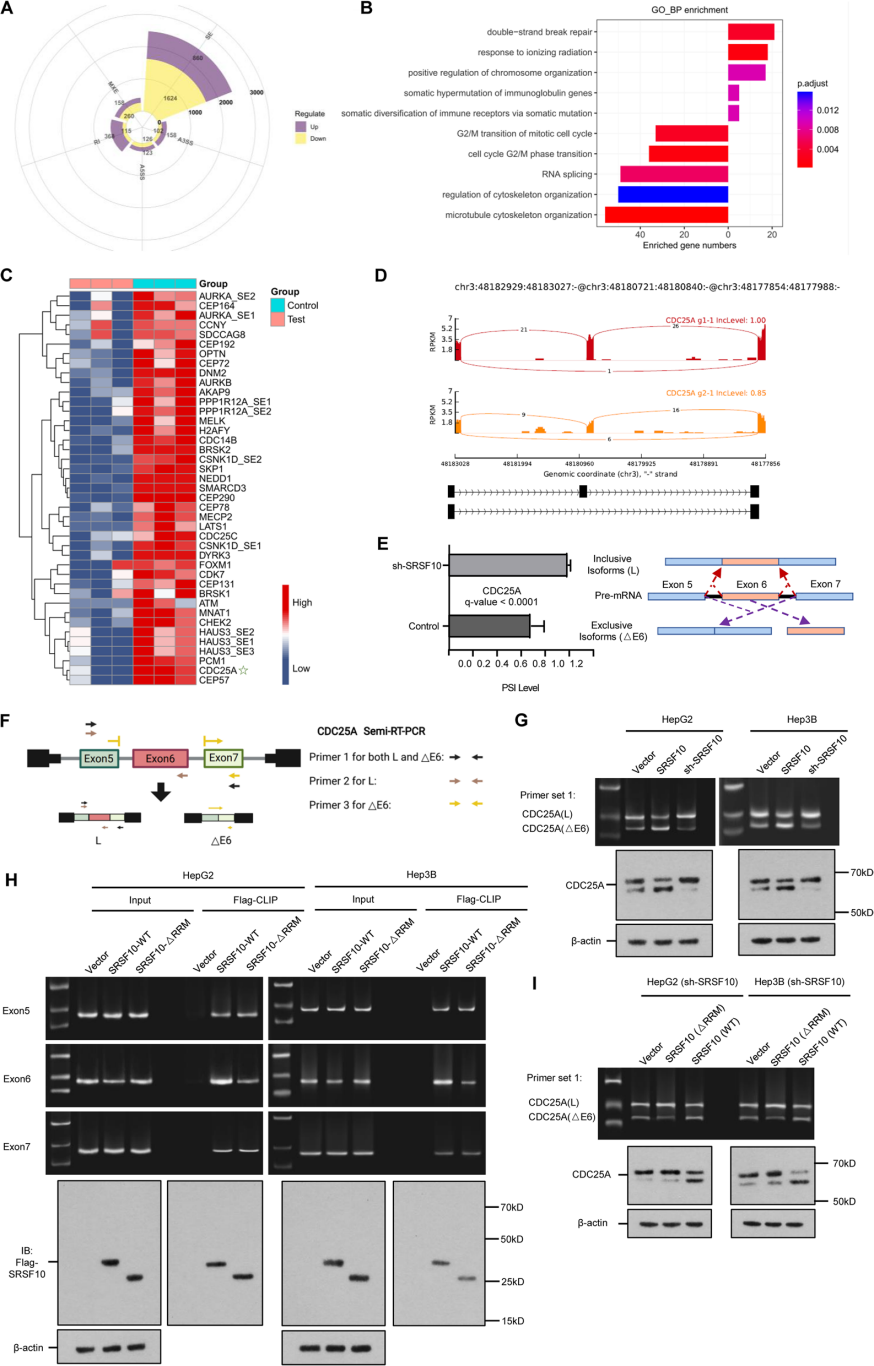
SRSF10 controls alternative splicing of pre-CDC25A through exon 6 skipping. **A** Pie graph depicting the alternative splicing events after splicing factor SRSF10 knockdown in Hep3B cells. The exon skipping accounted for large part of the AS events. **B** GO enrichment for potential targets of SRSF10. The top 5 upregulated and 5 downregulated pathways after SRSF10 knockdown are shown, which converged the AS events toward G2/M pathways. **C** Heatmap of cell-cycle related genes encompassing significantly changed exon skipping events under the SRSF10 silencing condition. CDC25A☆ ranked the highest and was selected as a potential target. All experiments were conducted in triplicate, and the results are presented as the log value (base 2) of the sh-SRSF10/control cell ratio. **D** Preliminary comparison of exon 6 skipping events between sh-SRSF10 and the control group. **E** Exon skipping in CDC25A measured in Hep3B cell line with stable knockdown of SRSF10 and the control. Left panel, mean ± SD of the percent-splice-in (PSI) from three experiments; the q value was calculated by paired Student’s t test. Right panel, diagram of exon 6 inclusive (Long, L) and exclusive (Exon 6 deleted, △E6) isoforms in the fate of pre-CDC25A splicing. **F** Schematic of three designed primers for the following semi-RT‒PCR. Primer set 1 was utilized to amplify all exon 6 inclusive and exclusive variants, while primer sets 2 and 3 were specific for only the L and △E6 isoforms individually, respectively. **G** Alternative splicing of CDC25A in exon 6 was examined by RT‒PCR (primer set 1) and western blot in two HCC cell lines. The enhanced CDC25A exon 6 missing isoform was verified under SRSF10 overexpression, while deleting SRSF10 resulted in long isoform retention. **H** Direct binding between the indicated SRSF10 protein and endogenous CDC25A RNA fragments was verified by CLIP-qPCR assay and Flag-western blotting detection. Flag-tagged SRSF10 wild type (WT) and motif RRM1 (11-84aa) deletion mutants (△RRM) were constructed, and the upper panel demonstrates that SRSF10 RRM1 deletion strikingly decreased its binding to exon 6. In the lower panel, immunoblotting with Flag-SRSF10 showed the ideal input of foreign SRSF10 protein, and displayed similar binding ability of SRSF10(WT) and SRSF10(△RRM) to endogenous CDC25A mRNA. **I** SRSF10 (△RRM) was unable to restore CDC25A exon 6 skipping. CDC25A exon 6 inclusive and exclusive isoforms and their corresponding proteins were detected under SRSF10 silencing in two HCC cell lines. The SRSF10(WT) substantially restored the endogenous SRSF10 pattern, whereas SRSF10 (△RRM) displayed no effects in exon 6 exclusion and remained responsive to SRSF10 deprivation

To study the effects of SRSF10 on HCC phenotypes, we next performed RNA-seq in Hep3B cells, which harbor high endogenous SRSF10 expression. The Gene Ontology (GO) term enrichment analysis of the RNA-seq data demonstrates that exon skipping (ES) was the most frequent AS event regulated by SRSF10 (Fig. 2A). Further analysis showed the top 5 upregulated and 5 downregulated pathways under SRSF10 depletion compared to the negative control. The overlapping genes in G2/M and mitotic processes drew our attention, highlighting a high degree of cell cycle genes accompanied by pertinent ES events (Fig. 2B). Intriguingly, CDC25A ranked top out of 31 cell cycle-related genes with exon skipping (Fig. 2C). CDC25A, a Cdc25 phosphatase family member, plays important roles in both the S-phase and the transition from G2 to mitosis progression by activating cyclin-dependent kinase-2 (CDK2) through dephosphorylation at threonine and tyrosine residues [23, 24]. Overexpression of CDC25A has been reported in multiple cancers such as HCC, and highly expressed CDC25A showed signs of poor prognosis (see Supplementary Fig. 4) [25]. Although the functions of CDC25A cell cycle-activating phosphatases are well acknowledged, their splicing patterns and subsequent contributions have rarely been uncovered in pancancer mechanisms. Based on the RNA-seq data, we explored the specific splicing sites of CDC25A regulated by SRSF10. It should be noted that the splicing variant with inclusion of exon 6 was significantly increased in SRSF10-depleted conditions. This finding is consistent with the two major CDC25A mRNA sequences based on the Ensembl datasets, namely, longer CDC25A(L) (ENST00000302506.8) and shorter CDC25A(△E6) (ENST00000351231.7) isoforms (Fig. 2D). The inclusion and exclusion of exon 6 in CDC25A indicates splicing transformation and varied percent-splice-in (PSI) values followed by SRSF10 (Fig. 2E).

The primers specific for both CDC25A(L) and CDC25A(△E6) were first utilized for HepG2 and Hep3B cell lines transfected with either SRSF10-overexpressing or sh-SRSF10 plasmids (Fig. 2F). The CDC25A(△E6) mRNA and protein bands were drastically increased upon SRSF10 overexpression but downregulated under sh-SRSF10 conditions in both cell lines (Fig. 2G). Similar to several of its SR siblings, the SRSF10 protein contains an RNA binding domain, also called the RNA recognition motif (RRM), that guides interactions with RNA and other protein components [26]. To further verify the regulatory effect of SRSF10 on the AS of pre-CDC25A, we generated a reporter plasmid containing a genomic DNA fragment of SRSF10(Flag-tagged) as well as a mutation deleting the 11–84 aa sequence lacking RRM named SRSF10(ΔRRM1). Specific primers for CDC25A exon 6 as well as its adjacent exons 5 and 7 were designed to test the potential binding of SRSF10 protein and CDC25A pre-mRNA using CLIP-qPCR assay. The results show that SRSF10(WT) led to a decreased exon 6 band, but SRSF10(ΔRRM1) protein failed to cause exon 6 exclusion. Exon 6 had a significantly higher affinity for SRSF10(WT) protein than the exon 5 and exon 7 regions, while fewer exon 6 bands were observed when immunoprecipitated with SRSF10 (ΔRRM1) protein. Immunoblotting with Flag-SRSF10 (bait protein) showed the ideal foreign SRSF10 protein level, which also verifies the direct binding between the indicated SRSF10 (corresponding to both WT and ΔRRM1) proteins and endogenous *CDC25A* RNA fragments (Fig. 2H). Additionally, in HCC cells with endogenous SRSF10 depletion, forced exogenous SRSF10(WT) could sustain CDC25A(△E6) abundance, whereas SRSF10(△RRM) displayed no effects on exon 6 exclusion and remained responsive to SRSF10 deprivation (Fig. 2I). The promoting role of SRSF10(WT) was much larger than that of SRSF10(△RRM) in terms of HCC growth and invasion (see Supplementary Fig. 5). These results clearly demonstrate that SRSF10 RRM is responsible for its essential recognition and subsequent splicing, primarily on exon 6 of pre-CDC25A.

### CDC25A(△E6) is highly expressed in HCC and has higher malignancy than the corresponding long isoform

**Fig. 3:**
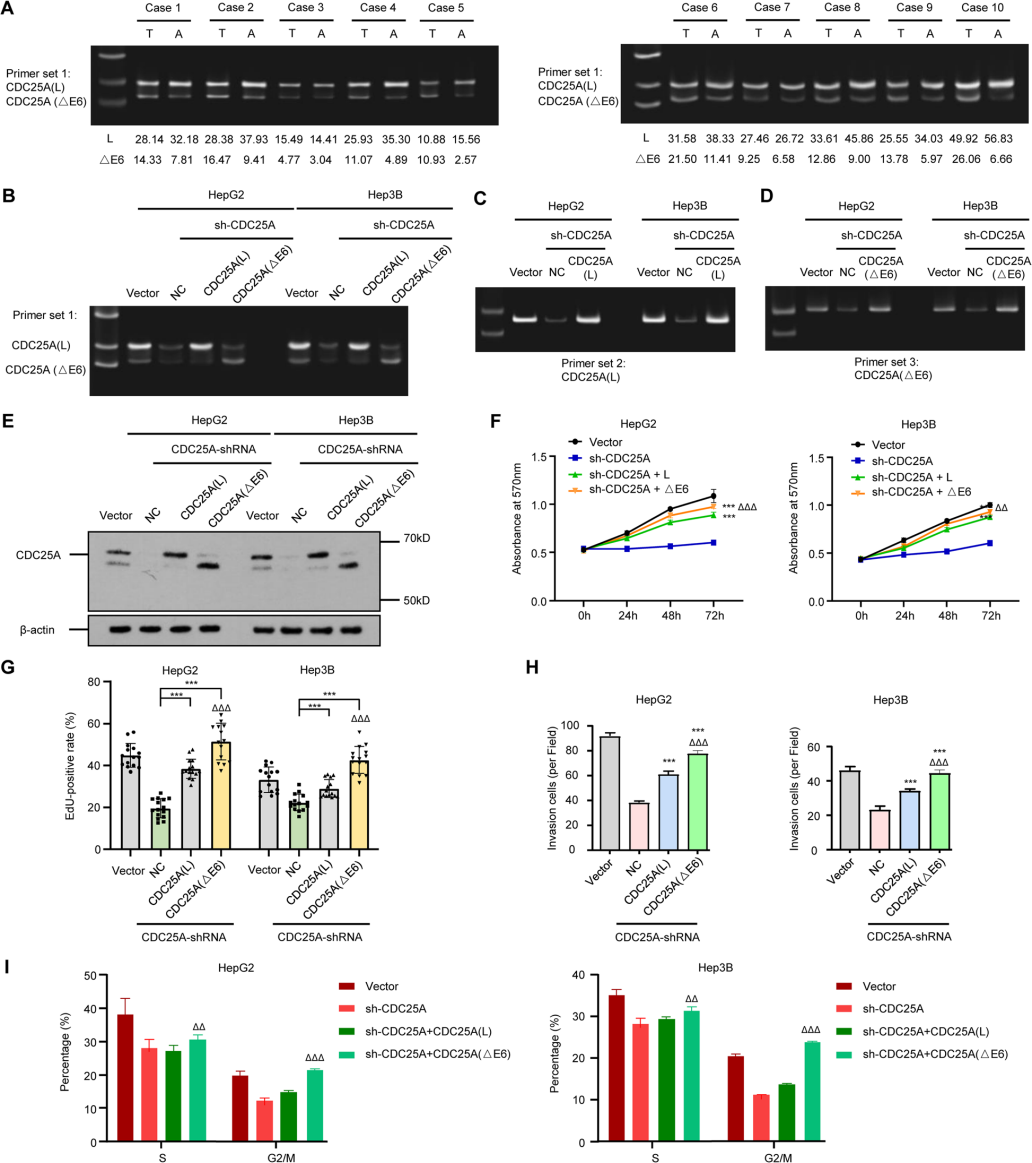
Abundant CDC25A(△E6) in HCC results in more malignant phenotypes than the CDC25A(L) isoform. **A** Expression of the CDC25A long and △E6 isoforms in HCC and adjacent nontumor tissue (10 pairs). The relative quantifications of CDC25A(L) and CDC25A(△E6) in each pair were shown underneath. **B**, **C**, **D**, **E** Restorative cell model validation of individual CDC25A(L) or CDC25A(△E6) following endogenous CDC25A deprivation by semi-RT‒PCR using three primer sets (**B**, **C**, **D**) and western blotting (**E**). **F**, **G**, **H**, **I** CCK-8, EdU, transwell assays, and flow cytometry showed the significantly higher promoting role of CDC25A(△E6) in cell viability (**F**), proliferation (**G**), invasion (**H**), and cell cycle (**I**) than CDC25A(L). Data are presented as the mean ± SD value of three biological replicates. ***p < 0.001 vs. NC, ^△△^*p* < 0.01 and ^△△△^*p* < 0.001 vs. CDC25A(L), t test. See representative images in Supplementary Fig. 7

CDC25A drives tumorigenesis of certain cancers, but the cell cycle ability of its splicing variants remains undocumented. Since our results suggest that CDC25A is prone to exclusive exon 6 by SRSF10, we sought to explore whether CDC25A(△E6) is critical for affecting malignancies in HCC cells. Stronger CDC25A(△E6) signals were observed in HCC tissues than in adjacent nontumor tissues in most of the 10 HCC cases, suggesting that highly expressed CDC25A(△E6) may have profound impacts on the biological effects of HCC cells (Fig. 3A). To study the different malignant phenotypes of CDC25A(△E6) and CDC25A(L), we deleted all endogenous CDC25A variants using full-length sh-CDC25A(FL), and the restoration of only CDC25A(△E6) or CDC25A(L) was verified by semi-RT–PCR (Fig. 3B, C, D using different primers) and western blot (Fig. 3E). As expected, silencing of endogenous CDC25A resulted in decreased cell viability, delayed DNA replication and mitotic progression, and reduced cell invasion ability (see Supplementary Fig. 6). In a series of restoration experiments, both CDC25A(△E6) and CDC25A(L) had positive effects on HCC cell proliferation, invasion, and cell cycle progression. However, CDC25A(△E6), not CDC25A(L), completely compensated for the inhibitory effect of CDC25A knockdown (Fig. 3F, G, H, I, and Supplementary Fig. 7). In a silencing experiment, knockdown of CDC25A(△E6) but not CDC25A(L) drastically inhibited cancer-promoting characteristics (see Supplementary Fig. 8). Our data further demonstrated that exogenous CDC25A(△E6) has significantly higher efficacy than CDC25A(L).

### Delineation of CDC25A stabilization and localization by ubiquitinating sites in exon 6

**Fig. 4:**
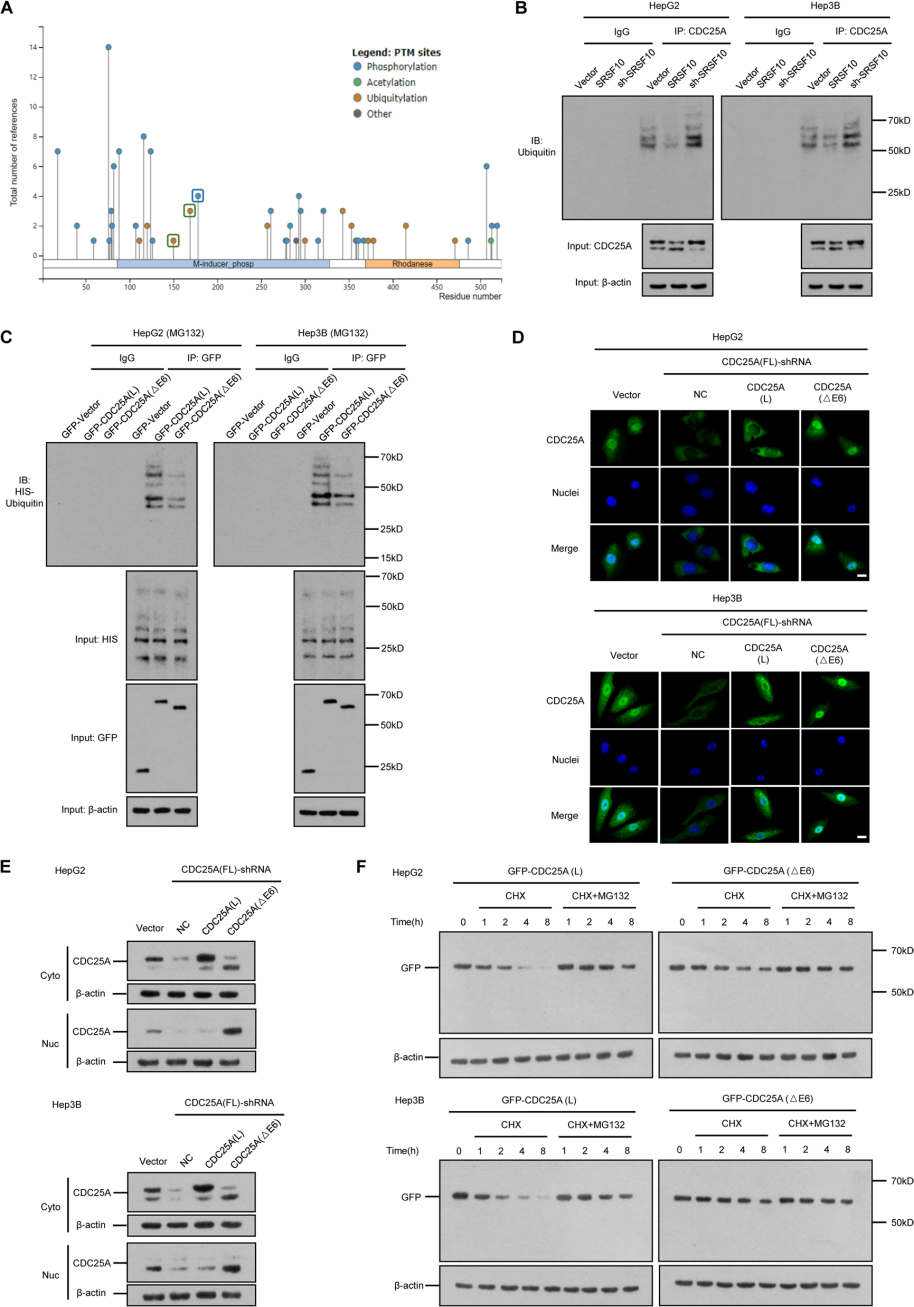
Exon 6 in CDC25A activates its ubiquitination and nuclear export. **A** The predicted ubiquitinating (K150, K169) and phosphorylating (S178) sites in exon 6 of CDC25A. Exon 6 resided in 144-183aa region, and PhosphoSitePlus (https://www.phosphosite.org/) was applied to predict the PTM sites. **B** SRSF10 led to less ub-CDC25A and more CDC25A(△E6) products. The total ubiquitination and input of CDC25A were examined by immunoprecipitation and western blotting, respectively. The shrinking of ub-CDC25A and higher CDC25A(△E6) expression were verified under SRSF10 overexpression, while deleting SRSF10 resulted in significantly higher ubiquitination levels and CDC25A(L) expression. **C** CDC25A(L) incorporating exon 6 resulted in a higher total ubiquitination level than CDC25A(△E6). The proteasome inhibitor MG132 was introduced in two HCC cell lines, and the ubiquitination levels were detected in GFP-tagged CDC25A(L) and CDC25A(△E6) conditions. The inputs of HIS and GFP indicated the changes in ub-CDC25A and specific total foreign CDC25A, respectively. **D, E** Subcellular localization comparison of CDC25A(L) and CDC25A(△E6). The immunofluorescence (**D)** and cell fractionation assays (**E**) verified that CDC25A(L) protein mainly resided in the cytoplasm while a significantly higher proportion of CDC25A(△E6) protein was retained in nucleus. Scale bars, 20 μm. **F** CDC25A(△E6) protein showed a prolonged half-life compared to CDC25A(L). Foreign GFP-tagged CDC25A(L) (C) and CDC25A(△E6) (D) were introduced to two HCC cell lines, and a protein stability assay was performed using CHX and MG132 at indicated time points

The high expression of CDC25A(△E6) and its greater tumorigenesis ability in HCC prompted us to examine the diverse regulatory mechanisms of proteins in comparison to CDC25A(△E6) and CDC25A(L). The significantly activated protein might depend on the seemingly “slight transformation” of exon 6 skipping. Since the CDC25A phosphatase domain (376–482 aa) did not reside in its exon 6 (144–183 aa) region, it is tempting to speculate that CDC25A function largely depends on the expression and stabilization of mRNA and/or protein. PTMs came to light since they have been identified to jointly control protein fates. We predicted the potential modified sites in CDC25A exon 6 sites, and selected two ubiquitinating sites and one phosphorylating site (Fig. 4A). These two processes for protein stability are being actively investigated and are believed to determine nuclear-cytoplasmic movement [27]. The regulatory patterns of CDC25A proteins following SRSF10 were then explored. We overexpressed and silenced SRSF10 in two HCC cell lines. The ubiquitination of endogenous CDC25A was detected on the basis of equal CDC25A loading in the IP panel. In accordance with our previous CDC25A protein expression tendency data, these results further demonstrated that SRSF10 led to less ub-CDC25A and more CDC25A(△E6) products (Fig. 4B). To further explore the causal relationships between the two CDC25A isoforms and ubiquitinating patterns, foreign CDC25A(△E6) and CDC25A(L)-overexpressing plasmids tagged with GFP were transfected into two HCC cell lines. Coimmunoprecipitation validated the significantly reduced ubiquitin levels of CDC25A(△E6) compared to CDC25A(L), suggesting that the ubiquitin-accelerating procedure was largely dependent on the existence of CDC25A exon 6 (Fig. 4C). Since the ubiquitinating process occurs in the cytoplasm, comparisons of the subcellular localization of CDC25A(L) and CDC25A(△E6) were conducted. Immunofluorescence and cell fractionation assays verified that CDC25A(L) protein mainly resided in the cytoplasm while a significantly higher proportion of CDC25A(△E6) protein was retained in the nucleus (Fig. 4D and E). A protein stability assay was further performed using CHX and MG132 reagents to determine the relevance of PTM sites on exon 6 and protein fate. The degradation of both isoforms was dependent on the ubiquitin–proteasome system (UPS), but relatively prolonged CDC25A(△E6) expression was detected compared to that of CDC25A(L) (Fig. 4F). The above data indicate that CDC25A lacking exon 6 by multiple factors, not limited to SRSF10, could possess nuclear retention, lower ubiquitin levels, and prolonged lifespan.

**Fig. 5:**
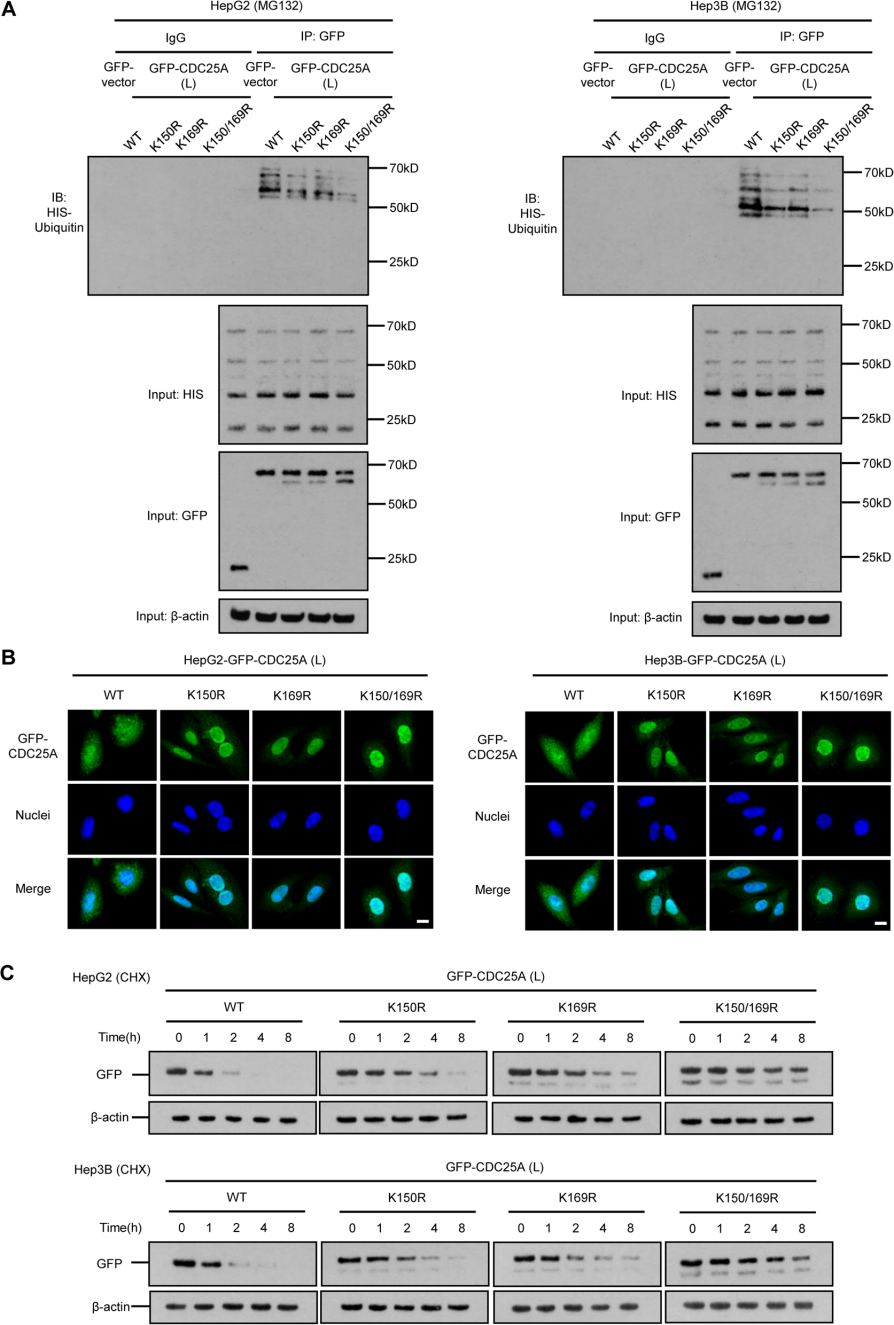
The lysine 150 and 169 sites in exon 6 affect total CDC25A ubiquitination and subcellular localization. **A** Both K150 and K169 were responsible for CDC25A ubiquitination. Single or double ubiquitin site mutations (K150R, K169R, and K150/169R) of CDC25A(L) were conducted. The immunoprecipitation results revealed less ubiquitination levels in both single-mutated situations, and double mutation further enhanced this effect. The HIS level showed no change of other proteins in these mutation systems, and the GFP input showed more CDC25A(△E6) expression in single- or double-mutated conditions. **B** Subcellular localization of the CDC25A(L) WT and mutated plasmids. Significantly stronger nucleus signals were detected in single- and double-mutated conditions. Scale bars, 20 μm. **C** Mutations in K150 and K169 enhanced foreign CDC25A(L) protein stability. HCC cell lines with WT and mutated GFP-tagged CDC25A(L) transfection were introduced into the CHX experiment at indicated time points

The transformation from CDC25A (L) to CDC25A(△E6) is easily detected as a consequence of the SRSF10-related splicing mechanism, but it is unclear whether ubiquitin in CDC25A-specific sites could individually determine its protein fate. Next, GFP-CDC25A(L) plasmids with single or double ubiquitin site mutations were transfected into HCC cells. Both K150 and K169 in exon 6 were responsible for CDC25A ubiquitination, and two locus mutations could drastically inhibit its process. Intriguingly, the input of GFP showed more CDC25A shorter isoform expression in these mutated conditions compared to wild-type conditions, and this splicing tendency toward shorter isoforms was more obvious in the double-mutated system. The above results might also hint at the close association among dynamic ubiquitination in exon 6, splicing isoforms (such as CDC25A(△E6)), and the corresponding protein stability (Fig. 5A). The localization experiments showed that CDC25A(L) protein mainly resided in the cytoplasm, while K150 and K169 mutated CDC25A(L) was retained in the nucleus (Fig. 5B). CHX experiments proved that mutation(s) in ubiquitin sites of exon 6 generated nuclei-enriched and stabilized CDC25A(L) (Fig. 5C). Additionally, the subtle CDC25A(△E6) and CDC25A(L) mRNA regulations were confirmed by an RNA stability assay, suggesting that inclusion and exclusion of exon 6 did not affect the half-life of the two CDC25A mRNA isoforms (see Supplementary Fig. 9). Given the working venue model that nucleus CDC25A functions in cell cycle progression while cytoplasmic CDC25A is prone to degradation [28, 29], we envision that CDC25A exon 6 skipping generated from SRSF10 not only restrains UPS-dependent protein degradation due to the absence of two ubiquitin sites but also accelerates its nuclear aggravation to enhance its cell cycle phosphatase activity.

### SRSF10 activates CDC25A by Ser178 dephosphorylation in exon 6

**Fig. 6:**
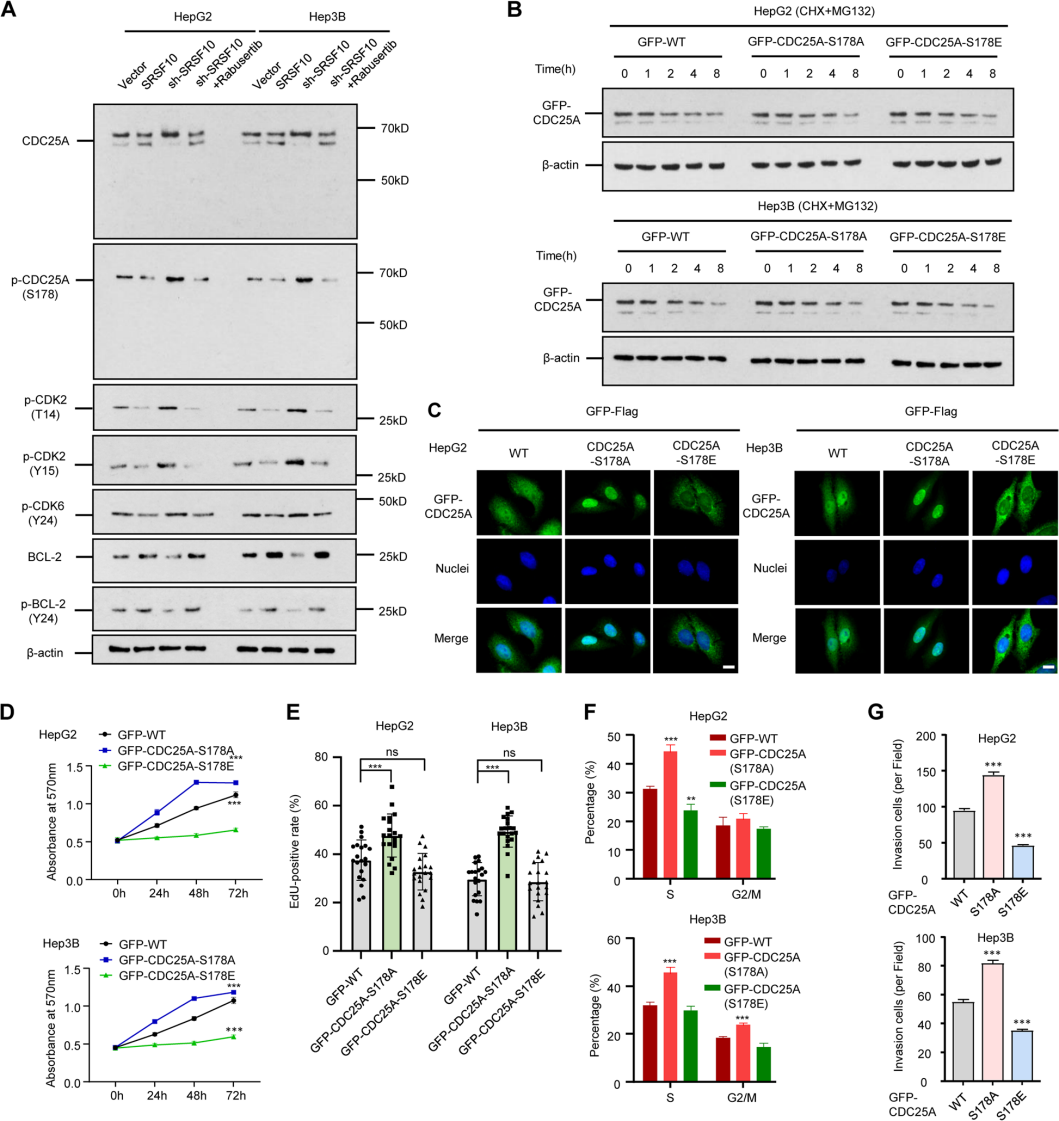
SRSF10 activates CDC25A through Ser178 dephosphorylation. **A** CDC25A associated cell cycle pathway and Ser178 phosphorylating status in response to SRSF10. Rabusertib is a highly selective CHK1 inhibitor that could restrain CDC25A phosphorylation. **B** Ser178 phosphorylation did not affect CDC25A stability. Ser178 substitution to alanine (A) suspended phosphorylation while glutamic acid (E) mutation could mimic constant phosphorylation. The protein stability assay was performed using CHX + MG132 intervention. **C** Ser178 phosphorylation affected CDC25A subcellular localization. Dephosphorylated CDC25A (S178A) was inclined to nuclear retention, whereas constant phosphorylated CDC25A (S178E) showed extranuclear abundance. Scale bars, 20 μm. **D**, **E**, **F**, **G** The dephosphorylation of CDC25A in Ser178 contributed to the cell viability (**D**), proliferation (**E**), cell cycle (**F**), and invasion (**G**) ability of HCC. Data are presented as the mean ± SD value of three biological replicates. **p < 0.01, ***p < 0.001 vs. WT, t test. See representative images in Supplementary Fig. 10

As a dual-specificity phosphatase, CDC25A removes inhibitory phosphorylation in cyclin-dependent kinases (CDKs) and positively regulates the activities of CDK4, CDK6, and CDK2 [30]. CDC25A protein levels vary in a cell cycle-dependent manner, with predominant expression in the G1 phase and stabilization in mitosis, which positively regulates cell cycle progression by helping pass the G1/S and G2/M checkpoints [31]. Moreover, CDC25A also switches from a steady state to a labile state during mitosis in a process regulated by phosphorylation [32]. Considering our previous finding that SRSF10 mediates CDC25A exon 6 skipping to trigger protein activity and stability, we next investigated whether SRSF10 affected multiple CDC25A phosphorylation sites, including Ser178 located at exon 6. The checkpoint protein CHK1 phosphorylates and degrades CDC25A to prevent the activation of CDK2 under DNA damage conditions. Rabusertib, a highly selective CHK1 inhibitor, was therefore introduced to HCC cells to restrain CDC25A phosphorylation [23, 33]. The double bands of CDC25A protein and single band of p-CDC25A(Ser178) showed skipped exon 6 by SRSF10, as expected. Given that Ser178 was the only potential phosphorylated site in the exon 6 region, it is reasonable to observe that p-CDC25A (Ser178) positively correlated with the upper band (exon 6 incorporated) of the CDC25A protein. Together with the subsequent results for p-CDK2, p-CDK6, p-BCL-2, and BCL-2, this finding preliminarily hints at the networks of downstream pathways and Ser178 phosphorylation driven by SRSF10 (Fig. 6A).

To evaluate the impact of this phosphorylation event on CDC25A properties, we generated full-length CDC25A versions substituting S178 with alanine (A) or glutamic acid (E), which lack phosphorylation or mimic constant phosphorylation, respectively. As CDC25A phosphorylation is often accompanied by proteasome-dependent degradation, we utilized CHX and MG132 experiments to determine whether CDC25A phosphorylation was modulated by this mechanism [34]. Mutation of Ser178 to alanine or glutamic acid did not change CDC25A protein stability (Fig. 6B). However, these mutations resulted in subcellular redistribution. The wild type was found in both the nucleus and the cytoplasm, while CDC25A-S178A was mostly nuclear, in contrast with the abundance of CDC25A-S178E in the cytoplasm (Fig. 6C). We assessed the effect of CDC25A S178 phosphorylation on the protein activity of each mutant. Mutation of S178 to alanine (A) significantly increased the cell proliferation, cell cycle, and invasion of HCC, while CDC25A-S178E had the opposite effect (Fig. 6D, E, F, G, and Supplementary Fig. 10). These results indicate that dephosphorylation at Ser178 fosters nuclear retention of CDC25A to promote HCC malignant types in response to SRSF10 effects. The increase in CDC25A(L) in the cytoplasm after S178 phosphorylation provides a shuttling mechanism between the two compartments, along with Ub-mediated degradation. In addition to increased CDC25A(△E6) and decreased CDC25A (L) by SRSF10, these results indicate how phosphorylation at Ser178 in exon 6 affects protein activity by nuclear-cytoplasmic redistribution.

### Exon 6 skipping of CDC25A is essential for SRSF10-mediated HCC malignant phenotypes

**Fig. 7:**
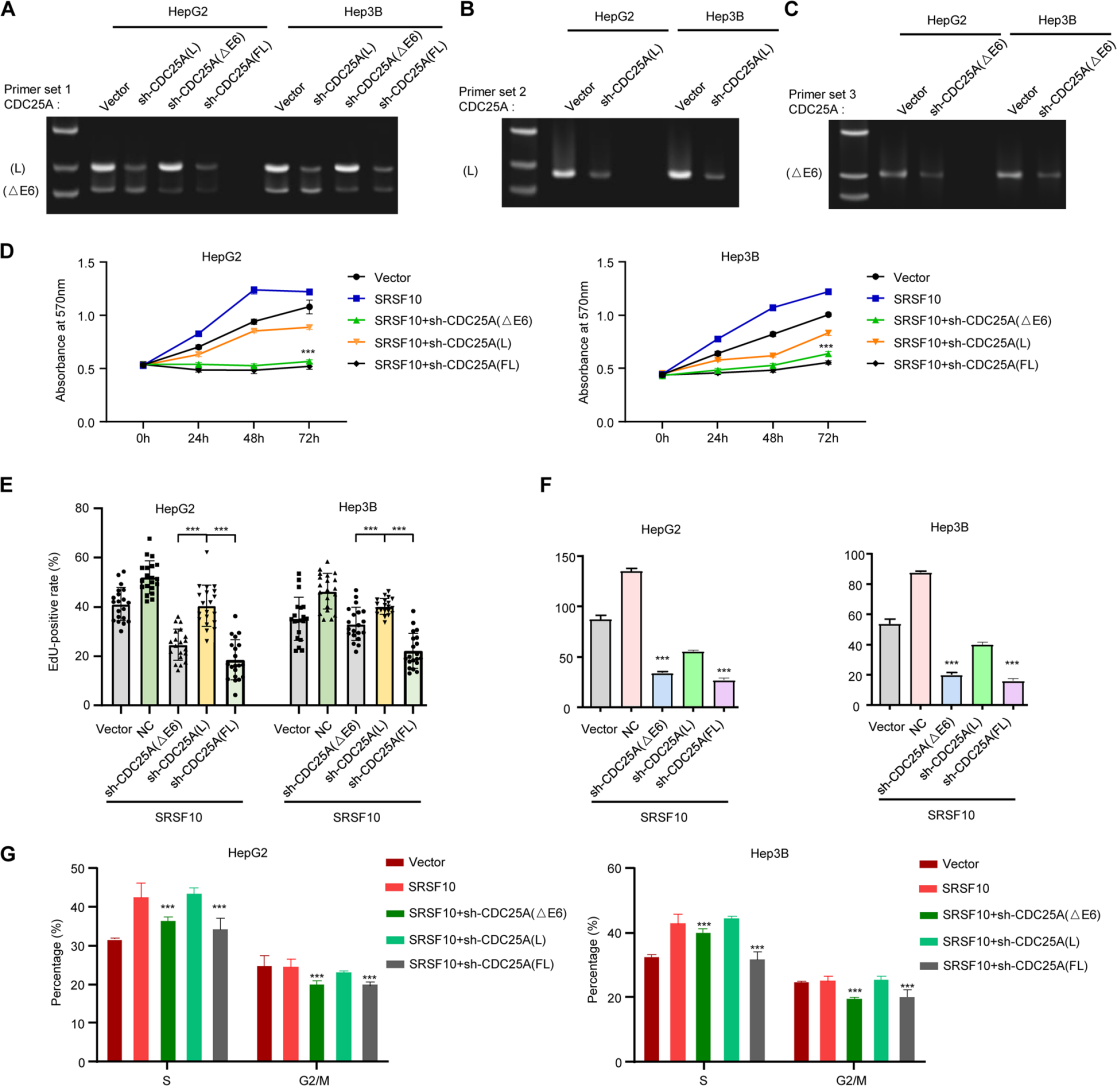
CDC25A(△E6) production is indispensable for SRSF10-mediated HCC progression in vitro and in vivo. **A**, **B**, **C** The validation of endogenous CDC25A(L), CDC25A(△E6), or all CDC25A(FL) knockdown by semi-RT‒PCR using three primer sets. **D**, **E**, **F**, **G** Effects of SRSF10 and diverse CDC25A variants on the cell viability (**D**), proliferation (**E**), invasion (**F**), and cell cycle (**G**) of HCC. Functional recovery experiments were conducted under silencing of CDC25A(L), CDC25A(△E6), and all endogenous CDC25A(FL) under circumstances of SRSF10 overexpression. Data are presented as the mean ± SD value of three biological replicates. ***p < 0.001 vs. sh-CDC25A(L), t test. See representative images in Supplementary Fig. 11

CDC25A lacking exon 6 contributes to protein stabilization and activation based on reduced ubiquitination and phosphorylation at specific sites, but whether SRSF10 is crucial for CDC25A-mediated aberrant cell cycle regulation still needs to be validated. Silencing of specific CDC25A (L), CDC25A (△E6), or all segments of CDC25A(FL) was employed to demonstrate the CDC25A exon 6 skipping-mediated effect (Fig. 7A, B, and C). Loss-of-function experiments were then conducted in the presence of SRSF10, revealing that CDC25A (△E6) and CDC25A (FL) depletion impeded cell viability and invasion to almost the same extent in the CCK-8 and transwell assays, while a partial decrease was found in the CDC25A (L) deletion group. Additionally, silencing CDC25A (△E6) had significant inhibitory effects compared with CDC25A (L) according to the EdU and cell cycle results (Fig. 7D, E, F, G, and Supplementary Fig. 11).

**Fig. 8:**
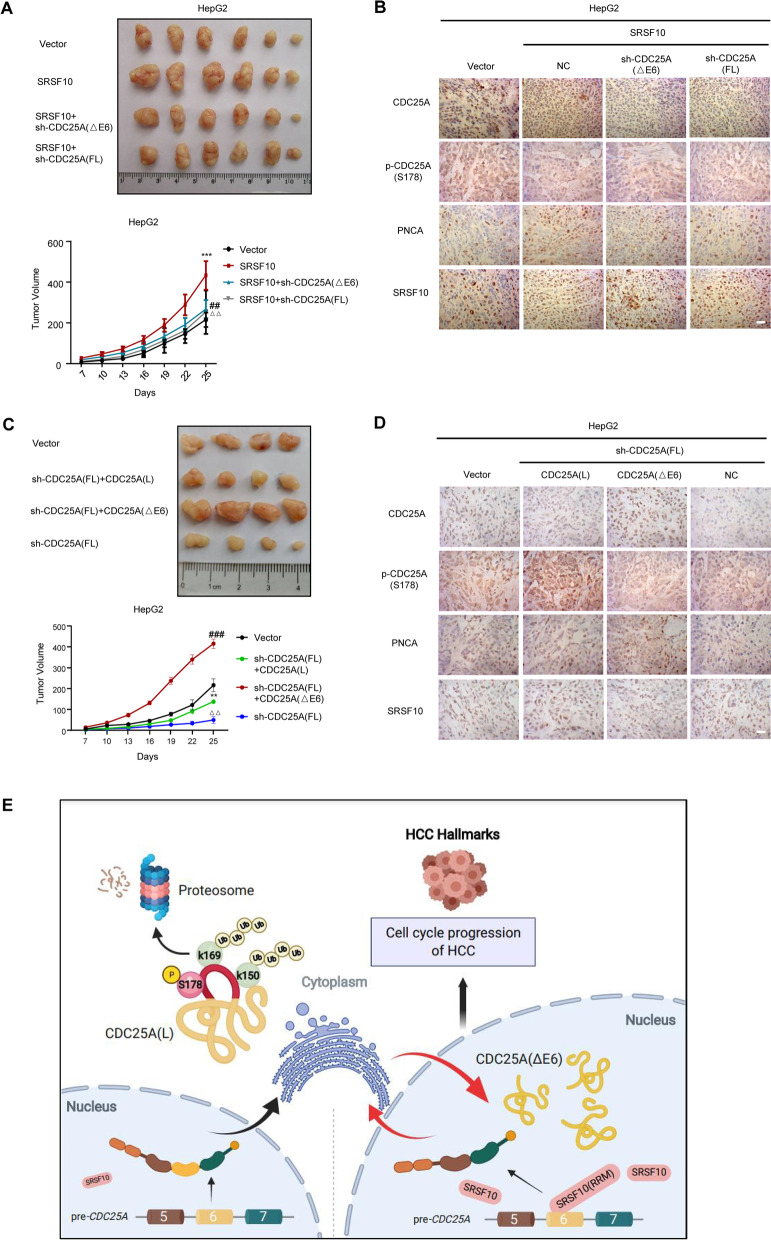
SRSF10 facilitates CDC25A(△E6) to promote HCC malignant phenotypes in vivo. **A** SRSF10 triggered HCC tumor growth, which was effectively counteracted by both sh-CDC25A(△E6) and CDC25A(FL) depletion. The size of HepG2 and Hep3B tumors formed in the subcutaneous implantation mice was monitored every three days. Data are presented as the mean ± SD values (*n* = 6). *SRSF10 vs. vector, ***p < 0.001; # SRSF10 + sh-CDC25A(△E6) vs. SRSF10, ##*p* < 0.01; ^△^SRSF10 + sh-CDC25A(FL) vs. SRSF10, ^△△^*p* < 0.01 (t test). **B** HCC tumorigenicity was confirmed by immunohistochemical staining of the isolated subcutaneous tumor tissue. SRSF10 dephosphorylated CDC25A at Ser178 and enhanced CDC25A expression. The results of low endogenous CDC25A expression in both sh-CDC25A(△E6) and sh-CDC25A(FL) groups indicate that SRSF10 contributed to a large proportion of CDC25A(△E6) production. Scale bars, 20 μm. **C** Foreign CDC25A (△E6) significantly enhanced tumor growth. Data are presented as the mean ± SD values (*n* = 4). *sh-CDC25A(FL) + CDC25A(L) vs. vector, **p < 0.01; # sh-CDC25A(FL) + CDC25A(△E6) vs. vector, ###*p* < 0.001; sh-CDC25A(FL) vs. SRSF10, △△*p* < 0.01 (t test). **D** Immunohistochemical staining of p-CDC25A(Ser178) and CDC25A in isolated subcutaneous tumor tissue. A large proportion of phosphorylated CDC25A(L) was observed in the cytoplasm. Foreign CDC25A(△E6) resided in nucleus while its Ser178 phosphorylated protein was hardly observed. **E** Schematic model of the SRSF10-dependent CDC25A exon 6 skipping event in HCC tumor progression. The working model shows that SRSF10 recognizes exon 6 of pre-CDC25A by its RRM domain, and the interaction of two molecules results in CDC25A exon 6 skipping. The production of the CDC25A(△E6) splicing isoform regulated by SRSF10 facilitates CDC25A protein stabilization by protecting it from nucleocytoplasmic transport and degradation, exerting its cell cycle properties to promote HCC progression

We next evaluated the in vivo behavior and phosphorylation status of CDC25A(L) and CDC25A (△E6) in response to SRSF10 using xenograft models of HepG2 cells. In agreement with the above data that SRSF10 acted as an HCC promoter, we found markedly larger tumor volumes accompanied by increased CDC25A and shrinking p-CDC25A(S178), and depletion of both CDC25A (△E6) and CDC25A (FL) effectively counteracted the promotion of tumorigenic ability in the presence of SRSF10, as anticipated (Fig. 8A and B). When endogenous CDC25A was depleted, foreign CDC25A (△E6) significantly enhanced tumor growth, while CDC25A(L) overexpression had a limited effect. Intriguingly, endogenous p-CDC25A(S178) staining was detected in both nucleus and cytoplasm, but a large proportion of phosphorylated CDC25A(L) was observed in the cytoplasm (Fig. 8C and D). Taken together, these observations consolidate the molecular mechanism of SRSF10-associated CDC25A (△E6) production and S178 dephosphorylation that is fundamental for hyperactivated and stabilized CDC25A protein in HCC progression both in vitro and in vivo (schematic diagram Fig. 8E).

## Discussion

Alternative splicing is the major mechanism of proteome diversification, and defects in core RNA processing have been recognized to promote cancer pathogenesis. As a well-established regulator of pre-mRNA alternative splicing and constitutive splicing, SRSF10 cooperates with c-Myc, TXNDC5, hnRNP A1/A2, and hnRNP F/H to form a splicing complex and regulate pre-mRNA transcription in multiple tumourigenes [35–38]. However, mechanistically, there are debates on whether SRSF10 acts as a splicing activator or suppressor. When identifying which splicing factors contribute to the exon skipping, the conserved motifs of SRSF10 is recently reported to recognize SREK1 exon 10 and facilitate its inclusion [39]. The direct role of SRSF10 in recognition and atypical splicing in HCC remains to be confirmed.

Most functional studies on human *srsf10* pre-mRNA production currently concern the main longer SRSF10-2 FL. It is highly conserved in mammals and is composed of three modules; the first module is an amino-terminal RRM that is responsible for directly binding to sequence motifs such as AGAGAV (V = A or G or C) or AAAGACAAA [40]. Meanwhile, two adjacent RS domains (RS1 and RS2) are located in the central and carboxy-terminal modules, and recent studies have revealed that the functions of RS1 and RS2 depend on cooperation with multiple RNA binding proteins (RBPs) [41, 42]. The RS domains together with RBP assembly in different contexts determine the diverse (activating or repressing) splicing roles, and intriguingly, RRM is unable to provide overall splicing activation if it is covalently linked to RS domain(s) in some cases. In contrast, only RRM helps SRSF10 recruitment to an exon and promotes its skipping [43, 44]. On the basis of a large proportion of exon skipping events in our RNA-seq results, we focused on the RRM regulatory patterns of SRSF10 to simplify the hypothetical model. Our results shed light on the novel role of SRSF10 in promoting the splicing of various pre-mRNAs, which is a major hallmark and potential driver for liver HCC. By combining in vivo and in vitro studies, we found that while constitutive mRNA splicing remains active, exon skipping events from SRSF10 generate mRNA isoforms in HCC cells.

In this study, we identified a large number of AS events through RNA sequencing following SRSF10, which were also preliminarily validated by our group. CDC25A is worthy of attention because its overexpression in cancer has frequently been associated with genetic instability and checkpoint abrogation, contributing to malignancy and a poor prognosis. CDC25A phosphatase plays a vital role in initiating the G1 to S phase transition via the cyclin E/Cdk2 complex [45]. Apart from its key role in controlling the cell cycle, CDC25A also functions as a mediator of checkpoint responses to DNA damage [46]. Paclitaxel induces cell cycle arrest at the G1 phase in response to DNA damage in association with the suppression of CDC25A expression. CDC25A protein stability relies on different ubiquitin ligase complexes (APC/C and Skp, Cullin, F-box (SCF)) through the ubiquitin–proteasome pathway [34, 47]. Our results provide a mechanistic understanding of proteasome-mediated CDC25A degradation followed by CDC25A exon 6 skipping.

Despite the documented role of CDC25A in cell cycle regulation, its expression and kinase activity are tightly controlled by phosphorylation events. We observed that the phosphorylation of CDC25A was also reduced by SRSF10 transfection. CDC25A was previously phosphorylated on specific residues, and its upstream kinases described to date are CHK1 and CHK2, p38 MAPK, CK1α, and NEK11 [34]. Site mutation experiments uncovered a unique role for CHK1-targeted CDC25A phosphorylation, and phosphorylation of serine 123, 178, 278, and 292 affects the turnover of CDC25A during interphase [48]. Another structural study identified serine 178 and threonine 507 residues phosphorylated by CHK1. However, mutation of T507 negatively regulates CDC25A activity, while S178 mutation alone does not impair its turnover. This discrepancy is due to domains within the N and C termini that are important for facilitating interactions between CDC25A and various cyclin/CDKs [49]. Phosphorylation on serine 283 increases the G2/M-promoting activity of CDC25A without impacting its stability or subcellular localization [50]. When evaluating the effect of rabusertib under SRSF10 silencing, it was beyond our expectation that the CHK1 inhibitor rabusertib not only dephosphorylated CDC25A at Ser178 but also restored exon 6 skipping, as shown in Fig. 6A. This interesting finding inspires us to further investigate whether Ser178 phosphorylation and the splicing of pre-CDC25A with elusive exon 6 are functionally linked in the future.

## Conclusions

In summary, we show that SRSF10 promotes exon 6 exclusion of CDC25A pre-mRNA splicing that yields the production of CDC25A(△E6). Importantly, CDC25A(△E6) is proven to be more stable, and the nucleus is retained due to the deletion of ubiquitinating and phosphorylating sites in exon 6, thus playing a stronger cell cycle role than CDC25A(L). Our work implicates dysregulated exon skipping in HCC and provides proof-of-concept evidence for a potentially appealing therapeutic target in this process.

## Supplementary Information

**Additional file 1:** **Table 1.** Primers and shRNA used in this study.

**Additional file 2:** **Table 2.** Antibodies for western blot, immunoprecipitation, immunofluorescence, and immunohistochemistry in this study.

**Additional file 3:** **Table 3.** Correlation between SRSF10 expression and clinicopathologic characteristics of the hepatocellular carcinoma patients. Percentage values are shown in parentheses.

**Additional file 4:** **Fig. 1.** Comparison of the SRSF10 mRNA level between HCC and adjacent nontumor tissue from the GEO database.

**Additional file 5:** **Fig. 2.** Immmunohistochemical staining of SIRT1 in HCC tissue and matching adjacent nonneoplastic hepatocyte tissue.

**Additional file 6:** **Fig. 3.** Correlation analysis between the five key genes and SRSF10 in HCC tissues.

**Additional file 7:** **Fig. 4.** The CDC25A mRNA level and its related survival curve in HCC patients from the NODE, GEO and TCGA databases.

**Additional file 8:** **Fig. 5.** Cell viability and invasion affected by wild-type and RRM-mutated SRSF10.

**Additional file 9:** **Fig. 6.** Identification of the effects of endogenous CDC25A and exogeneous full-length CDC25A on HCC biological functions.

**Additional file 10:** **Fig. 7.** Abundant CDC25A(△E6) in HCC results in more malignant phenotypes than the CDC25A(L) isoform.

**Additional file 11:** **Fig. 8.** Comparison of the effects of endogenous CDC25A(L), CDC25A(△E6) and CDC25A(FL) on HCC biological functions.

**Additional file 12:** **Fig. 9.** Measurement of the RNA stability of CDC25A by RT‒qPCR in presence of the transcriptional inhibitor Actinomycin D (ActD) at indicated time points.

**Additional file 13:** **Fig. 10.** SRSF10 activates CDC25A through Ser178 dephosphorylation.

**Additional file 14:** **Fig. 11.** CDC25A(△E6) production is indispensable for SRSF10-mediated HCC progression *in vitro* and *in vivo*.

## Data Availability

All data generated or analyzed during this study are included in this published article and its supplementary information files.

## Abbreviations

AS: Alternative splicing
ES: Exon skipping
HCC: Hepatocellular carcinoma
CDC25A: Cell division cycle 25 A
PSI: Percent splice-in
RIP: RNA immunoprecipitation
RNAseq: RNA sequencing
SR: Serine/arginine-rich proteins
SRSF10: Serine and arginine-rich splicing factor 10
UPS: Ubiquitin‒proteasome system
CLIP: Cross-linking immunoprecipitation
RRM: RNA recognition motif
PTM: Posttranslational modification
CHX: Cycloheximide
GSEA: Gene set enrichment analysis
