# Supplementary material for: SRSF10 stabilizes CDC25A by triggering exon 6 skipping to promote hepatocarcinogenesis
Source: J Exp Clin Cancer Res. 2022 Dec 20;41:353. doi: 10.1186/s13046-022-02558-0 (PMC9764681; doi:10.1186/s13046-022-02558-0)
Supplement: Supplementary file 1 — Additional file 1: Table 1. Primers and shRNA used in this study. [file 13046_2022_2558_MOESM1_ESM.docx]

**Supplemental Table 1** Primers and shRNA used in this study

| **Name** | **Forward/Sense** | **Reverse/Anti-sense** |
| --- | --- | --- |
| SRSF10-qPCR | TGAGGATGTTCGTGATGCTGA | CCTCCTTTCATAACTTCGGCTT |
| CDC25A exon 5-qPCR | TGGAGGTGAAGAACAACAGT | AGAATCAGAATGGCTCCTC |
| CDC25A exon 6 (primer 2) -qPCR | GCTCTGAAGAGGAGCCATTC | CAGAGTTCTGCCTCTGTGTGA |
| CDC25A exon 7-qPCR | TGAGGATGTTCGTGATGCTGA | CCTCCTTTCATAACTTCGGCTT |
| CDC25A primer 1-qPCR | TCGACCCAGATGAGAACAAG | AGCCATCATCCTCATCAGAC |
| CDC25A primer 3-qPCR | GAACAAGGAAAATCTTTCCTCA | ACAGGGAAGGGGAGTCAAAC |
| β-actin-qPCR | ACCCTGAAGTACCCCATCGAG | AGCACAGCCTGGATAGCAAC |
| 18S | CAGCCACCCGAGATTGAGCA | TAGTAGCGACGGGCGGTGTG |
| SRSF10-shRNA-1 | CACGCUAUGAUGAUUAUGATT | UCAUAAUCAUCAUAGCGUGTT |
| SRSF10-shRNA-2 | GCUGAAGACGCUUUACAUATT | UAUGUAAAGCGUCUUCAGCTT |
| SRSF10-shNC | UUCUCCGAACGUGUCACGUTT | ACGUGACACGUUCGGAGAATT |
| CDC25A(FL)-shRNA-1 | TAGACGTCCTCCGTCCATATC |  |
| CDC25A(FL)-shRNA-2 | TCAACTTCATCAATCACTTTA |  |
| CDC25A(L)-shRNA-1 | GAGTTTAAGAAGCCAGTAAGACC |  |
| CDC25A(L)-shRNA-2 | GGGTAAAGATCTCTTCACACAGA |  |
| CDC25A(△E6) -shRNA-1 | ACAAGGAAAATCTTTCCTCAAAT |  |
| CDC25A(△E6) -shRNA-2 | GAACAAGGAAAATCTTTCCTCAA |  |
