# Supplementary material for: SRSF10 stabilizes CDC25A by triggering exon 6 skipping to promote hepatocarcinogenesis
Source: J Exp Clin Cancer Res. 2022 Dec 20;41:353. doi: 10.1186/s13046-022-02558-0 (PMC9764681; doi:10.1186/s13046-022-02558-0)
Supplement: Supplementary file 2 — Additional file 2: Table 2. Antibodies for western blot, immunoprecipitation, immunofluorescence, and immunohistochemistry in this study. [file 13046_2022_2558_MOESM2_ESM.docx]

**Supplemental Table 2** Antibodies for western blot, immunoprecipitation, immunofluorescence, and immunohistochemistry in this study

| **Primary antibodies** | **Dilution** | **Company / Catalog** |
| --- | --- | --- |
| SRSF10 | 1:1000 (WB); 1:400 (IHC) | bioss, bs-13229R |
| CDC25A | 1:750 (WB); 1:100 (IP);  1:100 (IF); 1:100 (IHC) | Proteintech, 55031-1-AP |
| Ubiquitin | 1:200 (WB) | Proteintech, 10201-2-AP |
| p-CDC25A(S178) | 1:500 (WB); 1:200 (IHC) | LSbio, LS‑C358938 |
| p-CDK2(T14) | 1:2000 (WB) | Abcam, ab68265 |
| p-CDK2(Y15) | 1:1000 (WB) | Omnimabs, om637477 |
| p-CDK6(Y24) | 1:500 (WB) | Abcam, ab131469 |
| BCL2 | 1:1000 (WB) | Ptgcn, 12789-1-AP |
| p-BCL-2(T69) | 1:500 (WB) | bioss, bs-12578R |
| β-actin | 1:2000 (WB) | Ptgcn, 66009-1-Ig |
| PNCA | 1:1000 (IHC) | Proteintech, 10205-2-AP |
| Flag | 1:100 (IP); 1:500 (WB) | Sigma, F1804 |
| GFP | 1:1000 (WB); 1:100 (IP); 1:100 (IF); | Abcam, ab290 |
| His | 1:500 (WB) | Abcam, ab18184M |
| Human IgG | 1:150 (IP) | Bioss, #bs-0297P |
| GAPDH | 1:4000 (WB) | Abcam, ab125247 |
