# Supplementary material for: SRSF10 stabilizes CDC25A by triggering exon 6 skipping to promote hepatocarcinogenesis
Source: J Exp Clin Cancer Res. 2022 Dec 20;41:353. doi: 10.1186/s13046-022-02558-0 (PMC9764681; doi:10.1186/s13046-022-02558-0)
Supplement: Supplementary file 3 — Additional file 3: Table 3. Correlation between SRSF10 expression and clinicopathologic characteristics of the hepatocellular carcinoma patients. Percentage values are shown in parentheses. [file 13046_2022_2558_MOESM3_ESM.docx]

**Supplemental Table 3** Correlation between SRSF10 expression and clinicopathologic characteristics of the hepatocellular carcinoma patients. Percentage values are shown in parentheses.

| Variables | Total number | | SRSF10 expression (%) | | |
| --- | --- | --- | --- | --- | --- |
|  |  |  | Low expression (n = 24) | High expression (n = 50) | *P* value |
| Age (y) | |  |  |  | 0.911 |
| ≤53^a^ | | 36 | 10 (27.8) | 26 (72.2) |  |
| >53 | | 38 | 11 (28.9) | 27 (71.1) |  |
| Gender | |  |  |  | 0.527 |
| Male | | 64 | 19 (29.7) | 45 (70.3) |  |
| Female | | 10 | 2 (20.0) | 8 (80.0) |  |
| Tumor size (cm) | |  |  |  | 0.001 |
| ≤6.7^b^ | | 50 | 20 (40.0) | 30 (60.0) |  |
| ＞6.7 | | 24 | 1 (4.2) | 23 (95.8) |  |
| Tumor multiplicity | |  |  |  | 0.522 |
| Solitary | | 60 | 18 (30.0) | 42 (70.0) |  |
| Multiple | | 14 | 3 (21.4) | 11 (78.6) |  |
| Edmondson-Steiner grade | |  |  |  | 0.010 |
| I-II | | 55 | 20 (36.4) | 35 (63.6) |  |
| III-IV | | 19 | 1 (5.3) | 18 (94.7) |  |
| pT classification | |  |  |  | 0.062 |
| PT1/PT2 | | 56 | 19 (33.9) | 37 (66.1) |  |
| PT3/ PT4 | | 18 | 2 (11.1) | 16 (88.9) |  |

^a^median age. ^b^median size. ^c^Chi-square test.
