# Supplementary figures and images for: SRSF10 stabilizes CDC25A by triggering exon 6 skipping to promote hepatocarcinogenesis
Source: J Exp Clin Cancer Res. 2022 Dec 20;41:353. doi: 10.1186/s13046-022-02558-0 (PMC9764681; doi:10.1186/s13046-022-02558-0)

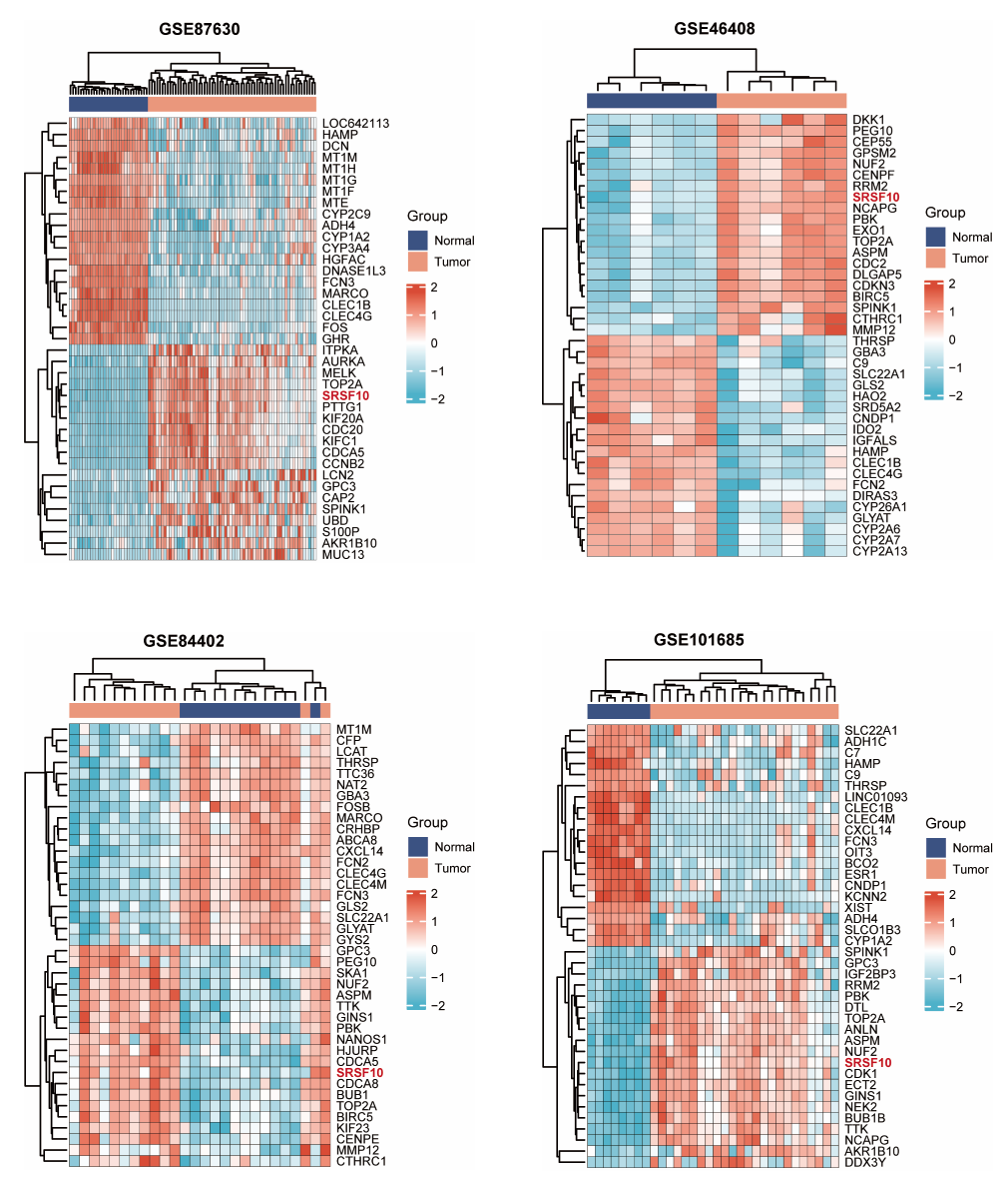

Supplement: Supplementary file 4 — Additional file 4: Fig. 1. Comparison of the SRSF10 mRNA level between HCC and adjacent nontumor tissue from the GEO database. [file 13046_2022_2558_MOESM4_ESM.tif]

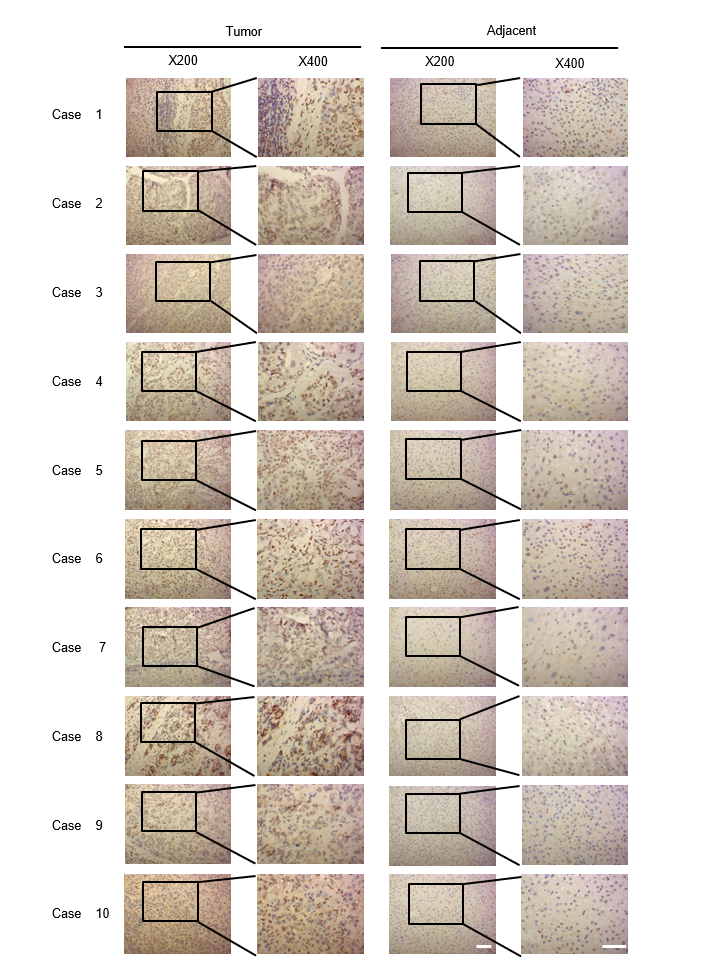

Supplement: Supplementary file 5 — Additional file 5: Fig. 2. Immmunohistochemical staining of SIRT1 in HCC tissue and matching adjacent nonneoplastic hepatocyte tissue. [file 13046_2022_2558_MOESM5_ESM.tif]

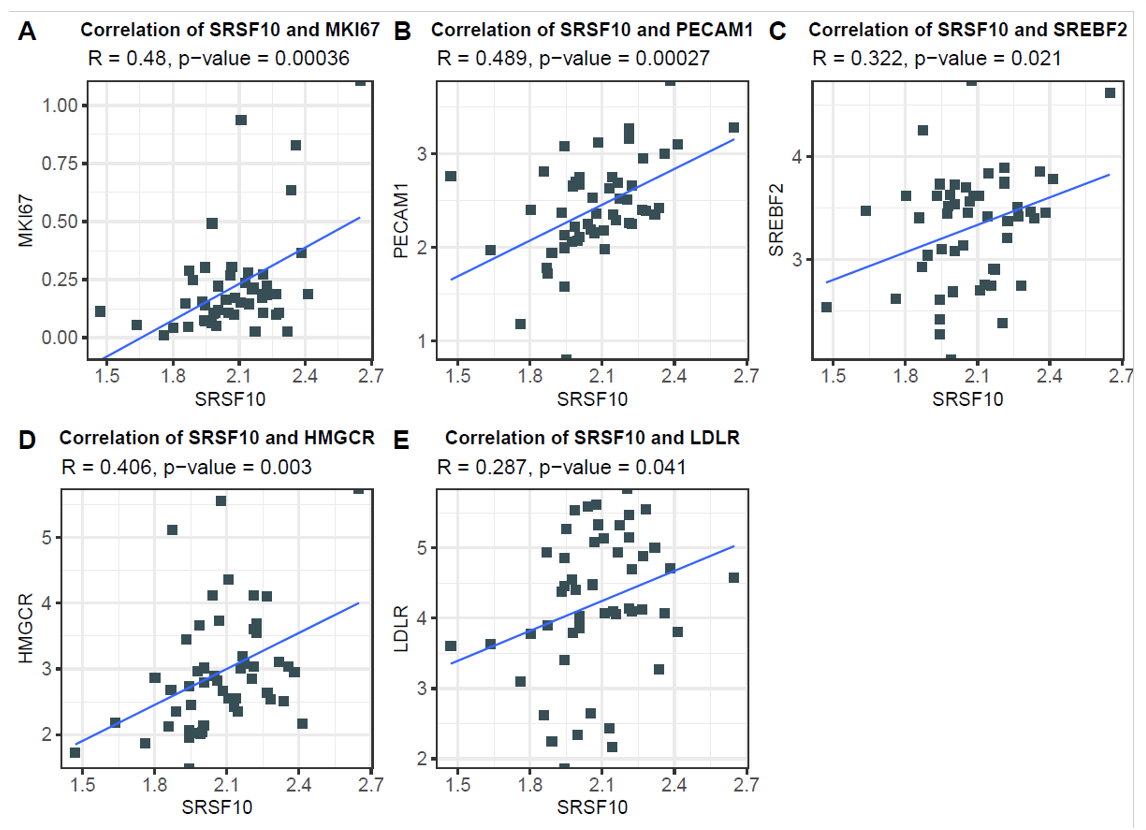

Supplement: Supplementary file 6 — Additional file 6: Fig. 3. Correlation analysis between the five key genes and SRSF10 in HCC tissues. [file 13046_2022_2558_MOESM6_ESM.tif]

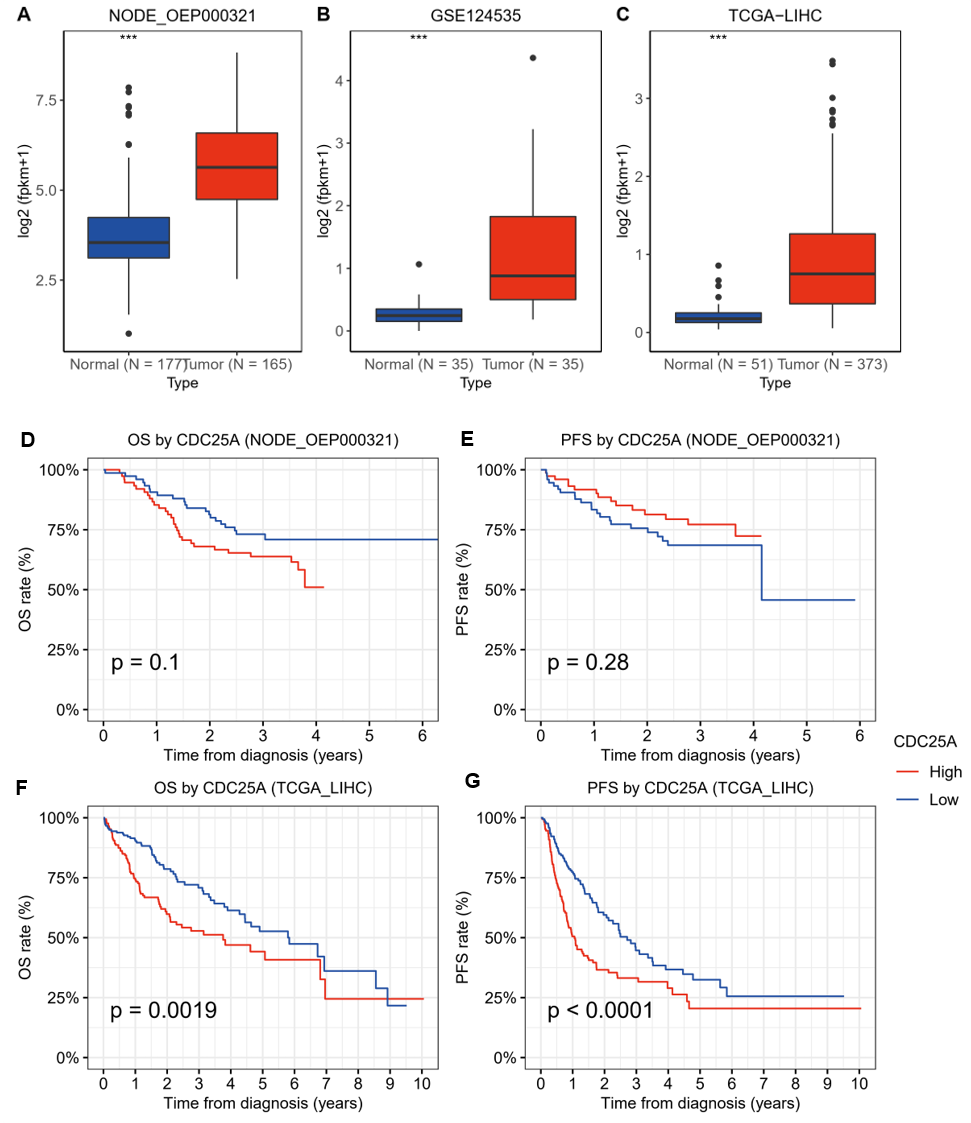

Supplement: Supplementary file 7 — Additional file 7: Fig. 4. The CDC25A mRNA level and its related survival curve in HCC patients from the NODE, GEO and TCGA databases. [file 13046_2022_2558_MOESM7_ESM.tif]

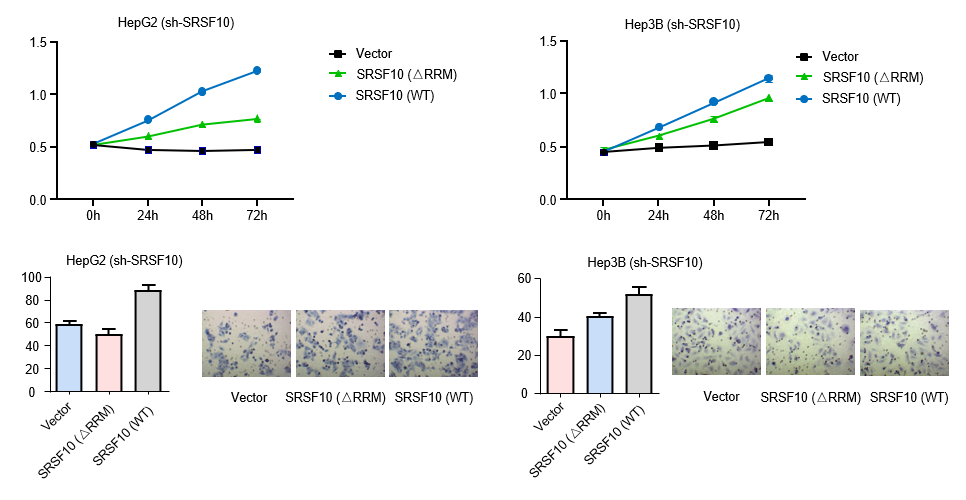

Supplement: Supplementary file 8 — Additional file 8: Fig. 5. Cell viability and invasion affected by wild-type and RRM-mutated SRSF10. [file 13046_2022_2558_MOESM8_ESM.tif]

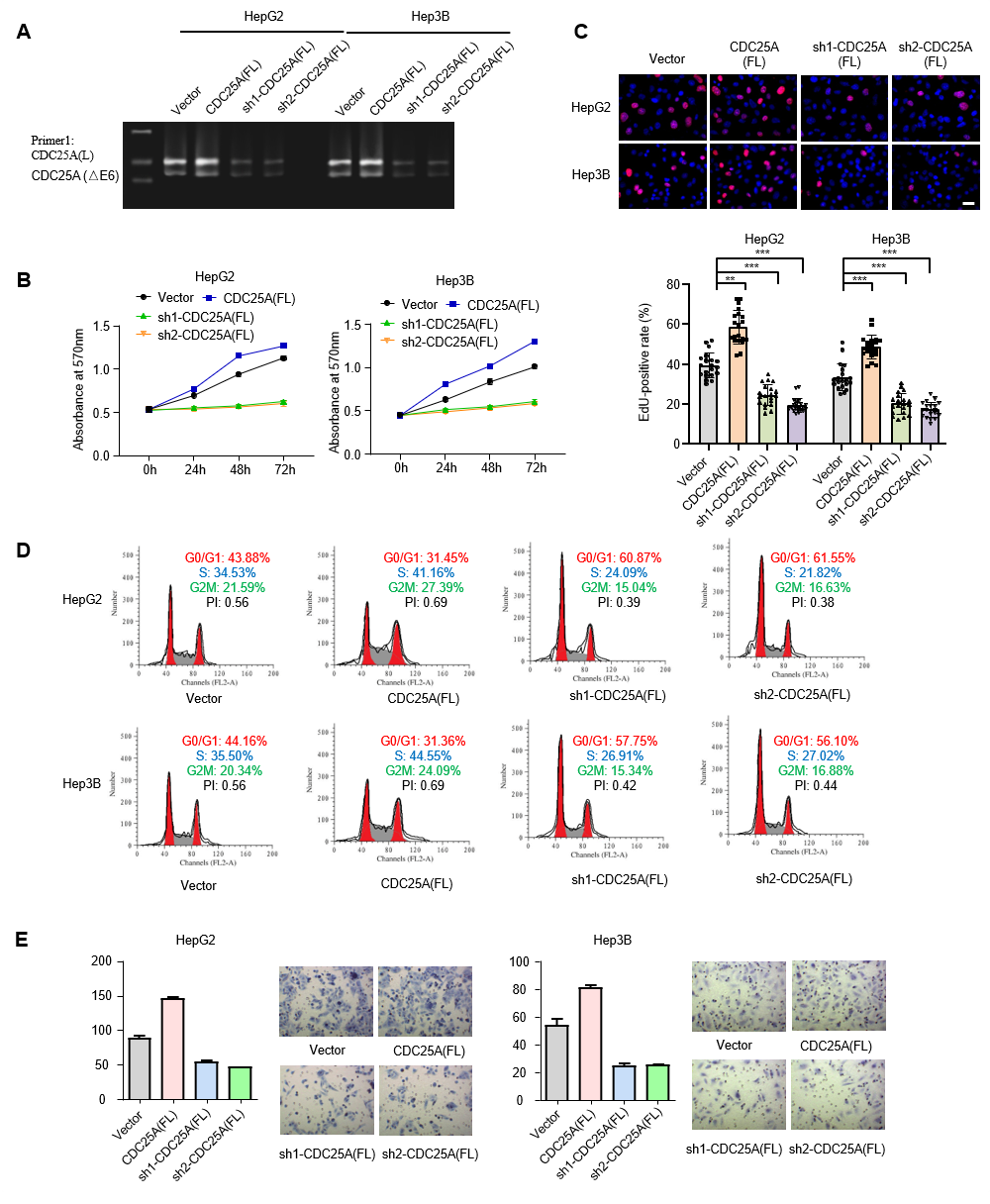

Supplement: Supplementary file 9 — Additional file 9: Fig. 6. Identification of the effects of endogenous CDC25A and exogeneous full-length CDC25A on HCC biological functions. [file 13046_2022_2558_MOESM9_ESM.tif]

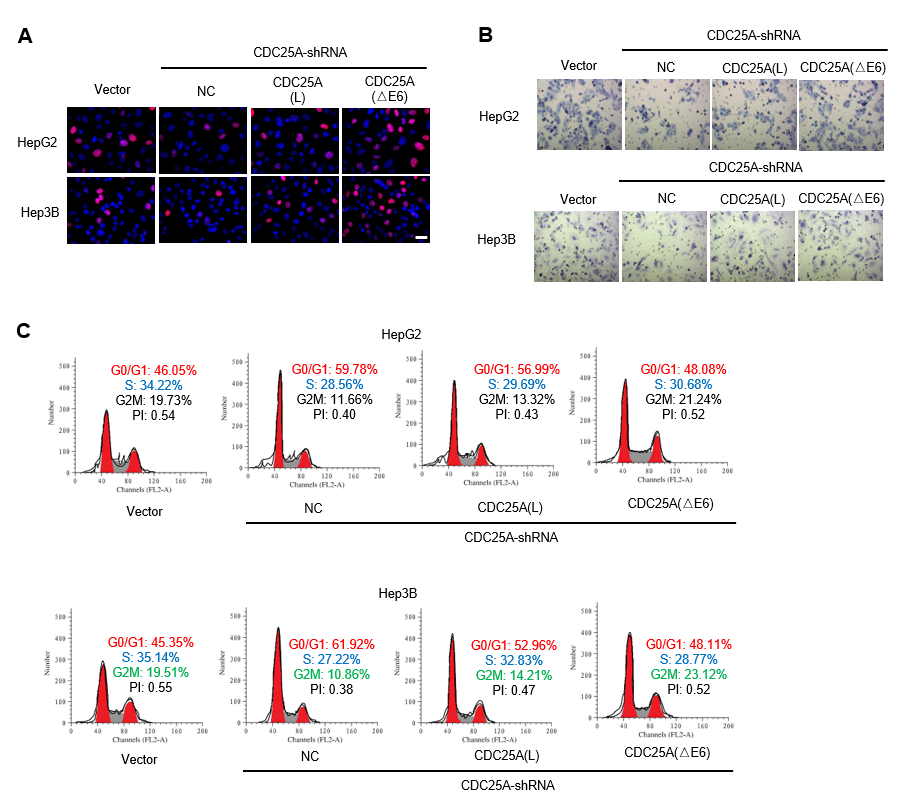

Supplement: Supplementary file 10 — Additional file 10: Fig. 7. Abundant CDC25A(△E6) in HCC results in more malignant phenotypes than the CDC25A(L) isoform. [file 13046_2022_2558_MOESM10_ESM.tif]

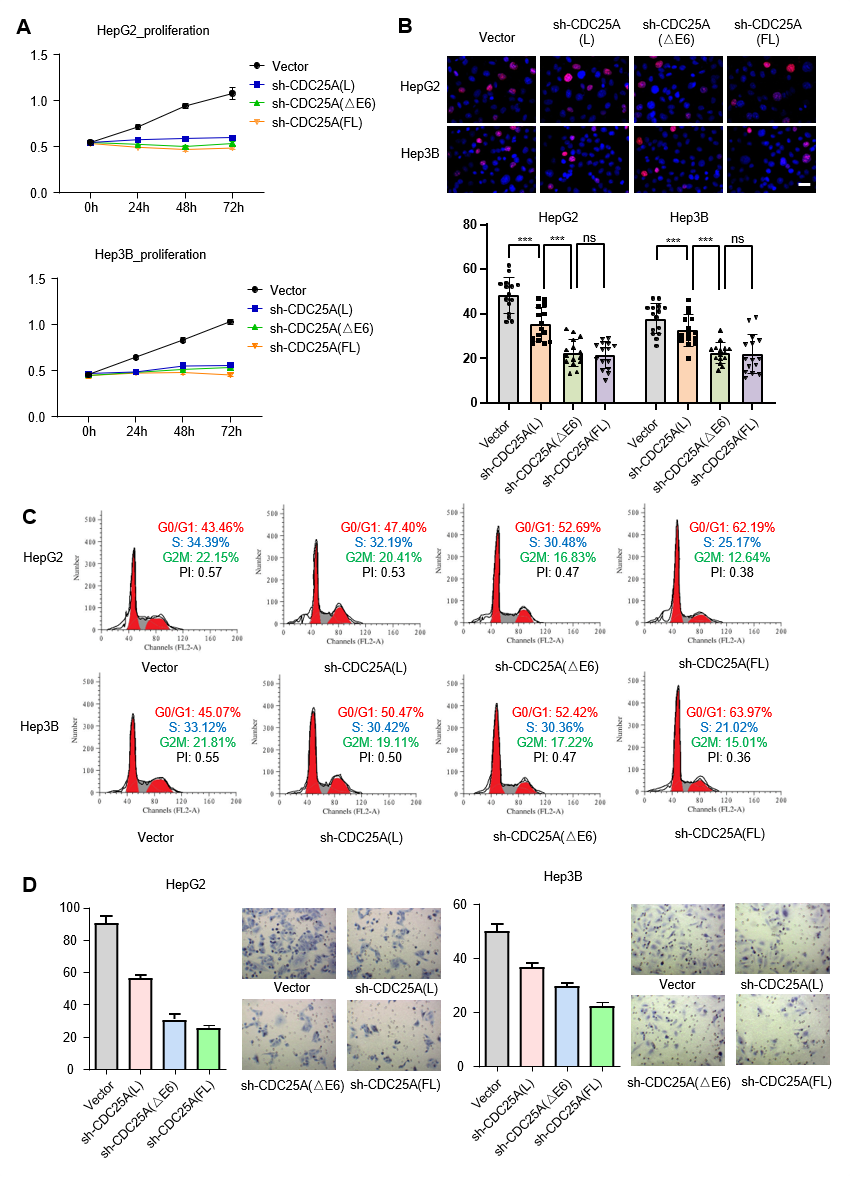

Supplement: Supplementary file 11 — Additional file 11: Fig. 8. Comparison of the effects of endogenous CDC25A(L), CDC25A(△E6) and CDC25A(FL) on HCC biological functions. [file 13046_2022_2558_MOESM11_ESM.tif]

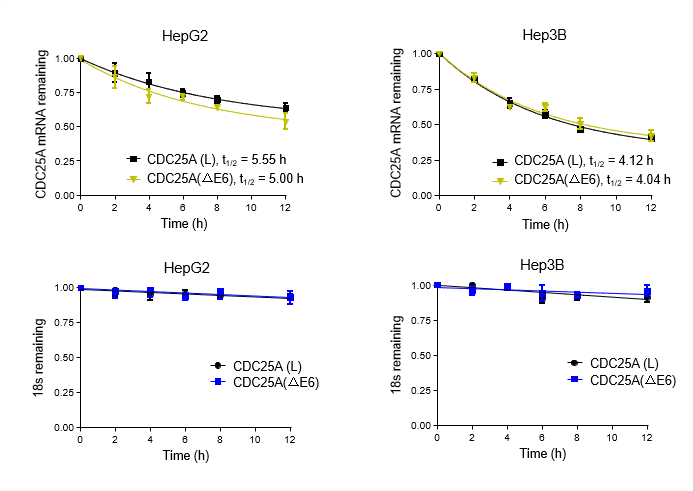

Supplement: Supplementary file 12 — Additional file 12: Fig. 9. Measurement of the RNA stability of CDC25A by RT‒qPCR in presence of the transcriptional inhibitor Actinomycin D (ActD) at indicated time points. [file 13046_2022_2558_MOESM12_ESM.tif]

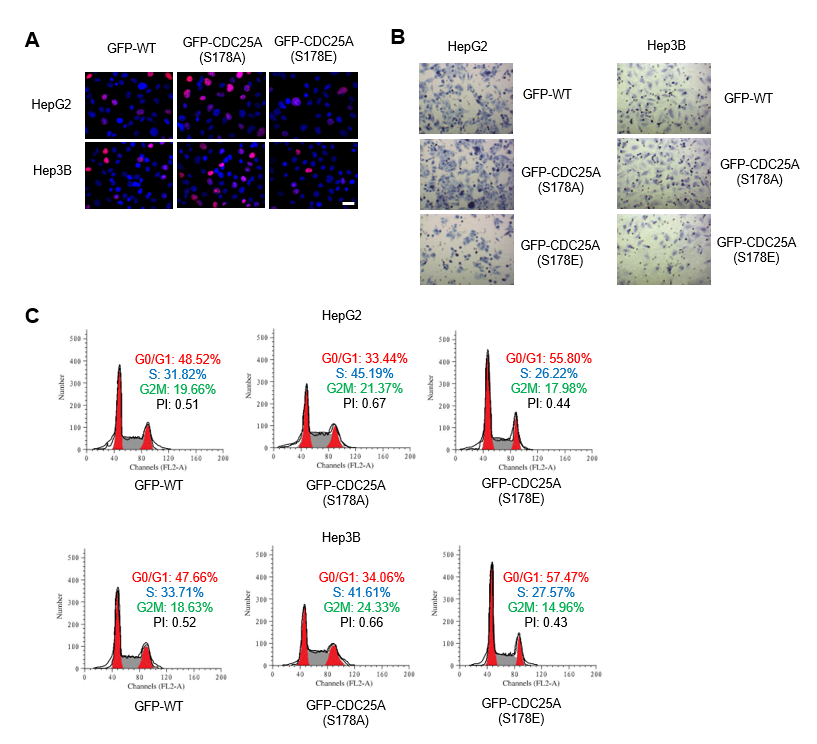

Supplement: Supplementary file 13 — Additional file 13: Fig. 10. SRSF10 activates CDC25A through Ser178 dephosphorylation. [file 13046_2022_2558_MOESM13_ESM.tif]

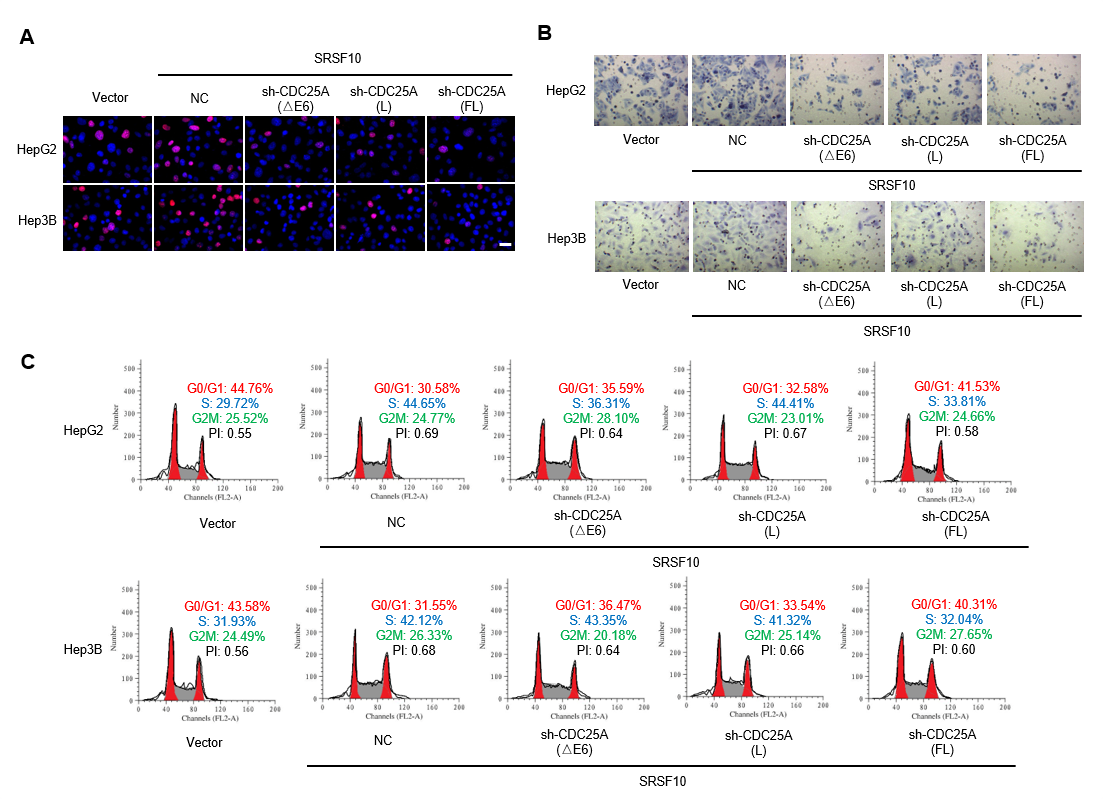

Supplement: Supplementary file 14 — Additional file 14: Fig. 11. CDC25A(△E6) production is indispensable for SRSF10-mediated HCC progression in vitro and in vivo. [file 13046_2022_2558_MOESM14_ESM.tif]
